# Strain-release alkylation of Asp12 enables mutant selective targeting of K-Ras-G12D

**DOI:** 10.1038/s41589-024-01565-w

**Published:** 2024-03-05

**Authors:** Qinheng Zheng, Ziyang Zhang, Keelan Z. Guiley, Kevan M. Shokat

**Affiliations:** 1 Department of Cellular and Molecular Pharmacology, Howard Hughes Medical Institute, University of California, San Francisco, CA USA; 2 Department of Chemistry, University of California, Berkeley, CA USA

**Keywords:** Small molecules, Cancer therapy, Cell signalling, Medicinal chemistry

## Abstract

K-Ras is the most commonly mutated oncogene in human cancer. The recently approved non-small cell lung cancer drugs sotorasib and adagrasib covalently capture an acquired cysteine in K-Ras-G12C mutation and lock it in a signaling-incompetent state. However, covalent inhibition of G12D, the most frequent K-Ras mutation particularly prevalent in pancreatic ductal adenocarcinoma, has remained elusive due to the lack of aspartate-targeting chemistry. Here we present a set of malolactone-based electrophiles that exploit ring strain to crosslink K-Ras-G12D at the mutant aspartate to form stable covalent complexes. Structural insights from X-ray crystallography and exploitation of the stereoelectronic requirements for attack of the electrophile allowed development of a substituted malolactone that resisted attack by aqueous buffer but rapidly crosslinked with the aspartate-12 of K-Ras in both GDP and GTP state. The GTP-state targeting allowed effective suppression of downstream signaling, and selective inhibition of K-Ras-G12D-driven cancer cell proliferation in vitro and xenograft growth in mice.

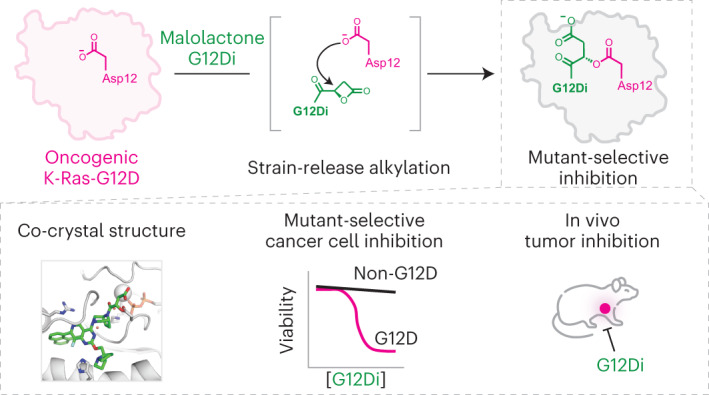

## Abstract (web summary)

Development of a malolactone electrophile that contains sufficient ring strain to counteract the weak nucleophilicity of aspartate enables covalent targeting of K-Ras-G12D, which is commonly found in pancreatic cancers.

## Main

Oncogenic mutations of Ras are among the most common genetic alterations in human cancer, with an estimated disease burden of >3 million new patients per year worldwide^1^. Despite widespread appreciation of the importance of Ras in cancer, direct binding ligands that block downstream signaling were not reported until 2013 (ref. ^2^) due to the lack of obvious drug-binding pockets in the protein. The only clinically approved K-Ras inhibitors are completely mutant specific as they rely on covalent recognition of the highly nucleophilic somatic cysteine residue of K-Ras-G12C. Recent preclinical reports^3,4^ of noncovalent K-Ras binding inhibitors have emerged that lack exclusive mutant specificity and exhibit varying degrees of biochemical preference for mutant K-Ras over the wild type. The challenge in covalent targeting the most common single variant in K-Ras-driven tumors (G12D) is that the carboxylic acid side chains of Asp and Glu are among the least nucleophilic heteroatom containing amino acid (a.a.) side chains. A further challenge for electrophiles capable of reacting with weak nucleophiles (Asp/Glu) is the need to withstand 55 M water and hypernucleophiles such as glutathione (GSH) in biological buffers. No suitable electrophiles for selectively targeting acidic a.a. side chains in aspartic or glutamic acids at physiological pH have been reported^5–8^ despite advances^9^ in targeting neutral or basic ones such as lysine^10–12^, tyrosine^13^, serine^14,15^, arginine^16^, methionine^17^ and histidine^18–20^. In this Article, we report an electrophile that alkylates the mutant aspartate in K-Ras-G12D in both GDP- and GTP-bound states, blocks effector interactions and inhibits the growth of K-Ras-G12D-driven cell lines.

## Results

### A malolactone covalently modifies mutant Aps12

**Extended Data Fig. 1:**
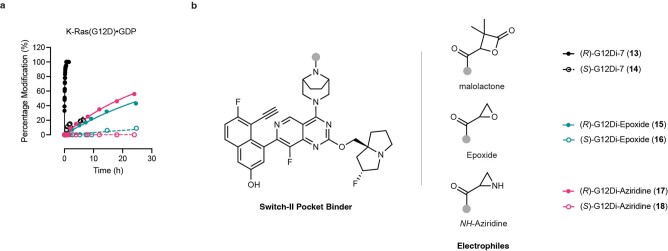
Covalent modification of K-Ras-G12D by strain-release electrophiles. **a**, Covalent labeling kinetics. Conditions: 200 nM K-Ras-G12D•GDP, 10 µM compound, 20 mM HEPES pH 7.5, 150 mM MgCl_2_, 23 °C. **b**, Chemical structures of strain-release electrophiles.

To covalently target the mutant aspartate, we focused on electrophiles designed to exploit strain release upon carboxylate attack of small three- and four-membered heterocyclic ring systems. Our^21^ and others’^22^ exploration of electrophiles from the literature showed modest levels of K-Ras-G12D crosslinking exhibited by three-membered rings—*NH*-aziridine and epoxide electrophiles covalently modified K-Ras-G12D by 40–60% over the course of 24 h (Extended Data Fig. 1a,b). Further optimization proved unsuccessful due to the limited number of chemically stable modifications possible in three-membered rings. A recent report^23^ demonstrated that structurally similar epoxides could label recombinant K-Ras-G12D at extremely high concentration (1 mM), which precluded its use as a targeted covalent inhibitor in cells. A covalent K-Ras-G12D inhibitor based on cyclophilin recruitment has been reported in a meeting abstract^8^.

**Extended Data Fig. 2:**
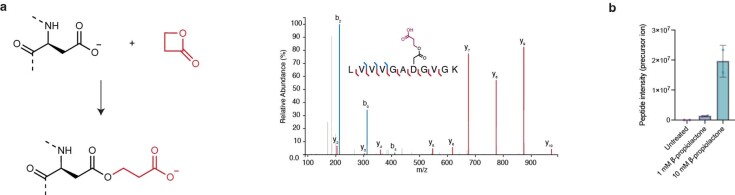
β-Propiolactone covalently labeled Asp12 of recombinant K-Ras-G12D. **a**, Alkylation of Asp12 by β-propiolactone and identified fragment ions of aa. 6-16 peptide including covalently modified Asp12. **b**, Extent of covalent modification is dose dependent. All data points represent individual biological replicates. Data are presented as mean ± SD (*n* = 2).

The four-membered ring electrophile β-lactone, found in both natural products and synthetic drugs^14,24,25^, was attractive because the parent β-propiolactone, a potent acylating and alkylating reagent^26–32^, modified K-Ras-G12D at multiple sites including the target Asp12 residue (Extended Data Fig. 2a) in a dose-dependent manner (Extended Data Fig. 2b). However, the [4.2.0]-fused β-lactones that covalently acylated a sibling K-Ras-G12S mutation^14^ were not reactive with G12D (Supplementary Fig. 1a), requiring exploration of another means to activate the nucleophilic attack of the β-lactone by Asp12.

Ambident electrophile β-lactones react with carboxylates with a preference at the β-carbon via an alkylation pathway at the β-carbon^25,33^ (cf. alcohols such as the serine side chain prefer acylation via carbonyl attack). The alkylation trajectory in the [4.2.0]-fused β-lactones at the bridgehead carbon was not accessible to Codon12 side chains (Supplementary Fig. 1b). We reasoned that switching the fused ring system to a simple carbonyl bridge would (1) geometrically enable the β-lactone electrophile to be poised for S_N_2 attack from the P-Loop Asp12, (2) activate the alkylation pathway by carbonyl π*-orbital participation and (3) potentially provide further hydrogen bonding activation by the neighboring Lys16 shown to be important in activating of K-Ras-G12C acrylamide electrophiles^34^.

**Fig. 1:**
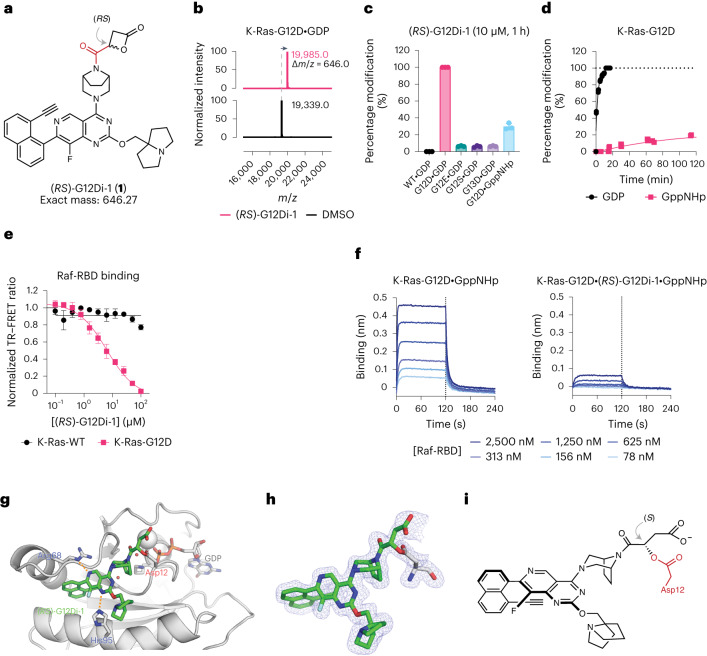
Malolactone (*RS*)-G12Di-1 (1) is a selective, covalent inhibitor of K-Ras-G12D. **a**, Chemical structure of racemic (*RS*)-G12Di-1. **b**, Deconvoluted protein mass spectra of Cyslight K-Ras-G12D•GDP in the presence or absence of (*RS*)-G12Di-1. Mass spectra are representative of two independent experiments. **c**, Compound (*RS*)-G12Di-1 selectively labeled K-Ras-G12D not WT or other mutants. All data points represent individual biological replicates. Data are presented as mean ± standard deviation (*n* = 3). **d**, Kinetics of K-Ras-G12D (200 nM) labeling with (*RS*)-G12Di-1 (10 µM) (*n* = 3, replicates are plotted as individual data points). **e**, Time-resolved fluorescence energy transfer dose–response of (*RS*)-G12Di-1 induced Ras-Raf•RBD binding disruption. All data points represent individual biological replicates. Data are presented as mean ± standard deviation (*n* = 3). **f**, Biolayer interferometry dose–response of GST-Raf•RBD binding with K-Ras-G12D•GppNHp without or with covalently labeled (*RS*)-G12Di-1. **g**, Co-crystal structure of K-Ras-G12D•GDP•(*RS*)-G12Di-1. **h**, 2*F*_o_–*F*_c_ map for the covalently bound ligand (*RS*)-G12Di-1 and Asp12 is depicted in blue mesh (1.0*σ*). **i**, Chemical structure of covalently bound ligand with the configuration of β-carbon assigned as *S*.

**Extended Data Fig. 3:**
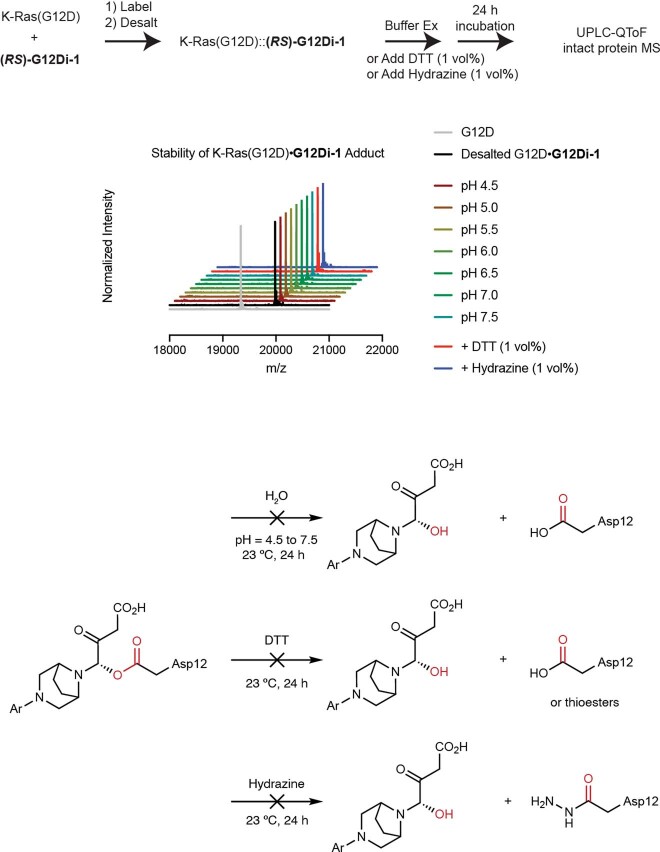
Covalent K-Ras-G12D•1 adduct is stable in pH 4.5–7.5 buffers and to thiol (DTT) or amine (hydrazine) electrophiles. Mass spectra are representative of two independent experiments.

A series of recently reported high-affinity noncovalent small molecules^35,36^, including MRTX1133 (refs. ^3,37^), which binds to K-Ras-G12D and wild-type (WT) K-Ras^38^ provided the necessary scaffold for presenting the electrophilic fragment toward the Asp12. We prepared (*RS*)-G12Di-1 (**1**) as a racemate by coupling (±)-β-carboxyl-β-propiolactone, also known as malolactone, to the bicyclic piperazine group of a Switch-II Pocket (S-IIP) ligand scaffold using the carbonyl bridge design (Fig. 1a). Using whole-protein mass spectrometry (MS), we assessed the reactions between recombinant K-Ras proteins and 10 µM (*RS*)-G12Di-1 at 23 °C (Fig. 1b,c). (*RS*)-G12Di-1 reacted rapidly with K-Ras-G12D•GDP with a half-life of 99 s (95% confidence interval 83–118 s) and fully modified K-Ras-G12D•GDP in less than 15 min (Fig. 1d). By contrast, (*RS*)-G12Di-1 achieved only 6.5 ± 0.3% modification of K-Ras-G13D•GDP and no detectable modification of K-Ras-WT•GDP after 1 h. Interestingly, (*RS*)-G12Di-1 showed markedly reduced reactivity with K-Ras-G12E•GDP, a nonnatural mutant that positions its carboxylate nucleophile (Glu) further from the backbone than that in G12D, and K-Ras-G12S•GDP. Although less potent, (*RS*)-G12Di-1 was also active against the GppNHp-bound K-Ras-G12D. This is distinct from S-IIP K-Ras-G12C inhibitors, which exclusively recognize the GDP-bound state^2,34,39–42^, whereas GTP-state recognition has so far only been observed for compounds with a different binding mechanism^8,43^. A recently reported K-Ras-G12C inhibitor dependent on cyclophilin recruitment is capable of GTP-state recognition and showed faster in-cell signaling inhibition kinetics^43^. The covalent modification of Asp12 by (*RS*)-G12Di-1 stabilized both K-Ras-G12D•GDP and K-Ras-G12D•GppNHp toward thermal denaturation (Δ*T*_m_ = +10.3 °C and +2.5 °C, respectively). The adduct between K-Ras-G12D and (*RS*)-G12Di-1 was stable over pH 4.5–7.5, and did not degrade in the presence of 1 vol% of dithiothreitol (DTT) or hydrazine at 23 °C showing its intrinsic resistance to nucleophiles in the bulk solution (Extended Data Fig. 3).

The ability of (*RS*)-G12Di-1 to engage the GppNHp-bound state of K-Ras-G12D prompted us to ask whether such a covalent modification could disrupt the interaction with effector proteins such as Raf. Using a time-resolved fluorescence energy transfer assay^44^, we found that (*RS*)-G12Di-1 inhibited the interaction between Raf-RBD and K-Ras-G12D, but not WT K-Ras, in a dose-dependent fashion (Fig. 1e). We also measured the interaction between immobilized Raf-RBD with fully labeled K-Ras-G12D•GppNHp•(*RS*)-G12Di-1 using biolayer interferometry. Compared to unmodified K-Ras-G12D•GppNHp, the K-Ras-G12D•GppNHp•(*RS*)-G12Di-1 complex showed substantially decreased binding to Raf-RBD (Fig. 1f). Such a feature may be particularly advantageous for the G12D mutant, as its severely impaired GTP hydrolysis^45^ renders an abundant and persistent K-Ras population in the active GTP-state in the cell.

### Mechanism- and structure-guided ligand evolution

**Extended Data Fig. 4:**
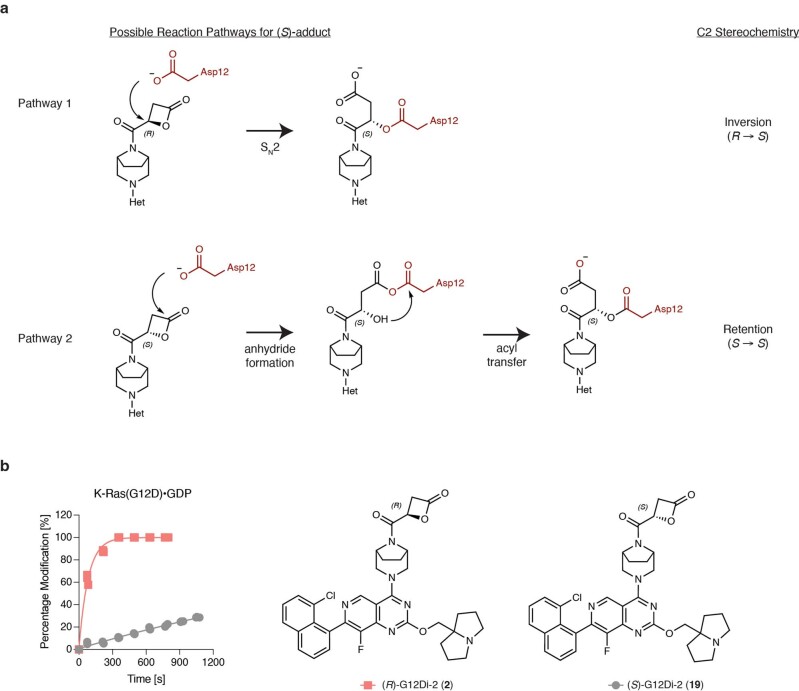
Mechanistic rationale of the enantioselectivity of the covalent modification reaction. **a**, Two possible reaction pathways to form (*S*)-adduct with distinct stereochemical events at α-carbon. **b**, K-Ras-G12D favors an (*R*)-enantiomer of the malolactone electrophile.

To better understand the covalent reaction in the S-IIP of KRAS (G12D) between (*RS*)-G12Di-1 and Asp12, we solved a 1.7-Å crystal structure of the K-Ras-G12D•GDP•(*RS*)-G12Di-1 adduct (Fig. 1g). We observed (*RS*)-G12Di-1 in the S-IIP pocket adopting a conformation similar to that seen for K-Ras-G12C inhibitors (Supplementary Fig. 2), with clear electron density for the covalent ester bond between Asp12 and the compound as well as the free carboxyl group resulting from the ring opening. This high-resolution structure also allowed us to assign the stereochemistry of the adduct as *S* at the β-carbon (Fig. 1h,i). Because we obtained the co-crystal using racemic (*RS*)-G12Di-1, this *S* stereochemistry could in theory result from an S_N_2 attack on the β-carbon of *R* enantiomer of G12Di-1 or an attack on the carbonyl of the *S* enantiomer of G12Di-1 followed by an acyl transfer (Extended Data Fig. 4a). To distinguish between these two possibilities, we prepared pure *R* and *S* enantiomers of a structural analog. Monitoring the reaction rate with K-Ras-G12D•GDP showed that the *R* enantiomer (*R*)-G12Di-2 (**2**) was significantly more reactive toward K-Ras-G12D•GDP (Extended Data Fig. 4b). Furthermore, we observed the same *R* » *S* C_α_-enantioselectivity (that is, C_2_ in malolactone system) in the context of K-Ras-G12D labeling across a panel of strain-release electrophiles such as epoxides and aziridines that can only react via an S_N_2 mechanism (Extended Data Fig. 1). Such enantioselectivity favoring a direct S_N_2 ring-opening mechanism aligns with literature observations with β-lactones^25,33^. The predominant S_N_2 preference of ring-opening reaction by Asp12 is distinct from the preference for attack by water, that is, hydrolysis, which often takes place at the carbonyl in near-neutral buffers^46^.

**Fig. 2:**
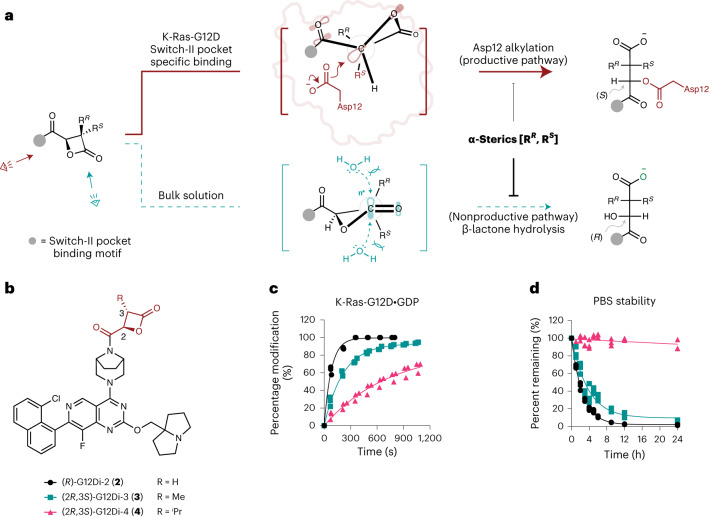
Sterically hindered β-lactones are stable and potent inhibitors of K-Ras-G12D. **a**, Mechanistic analysis of the Asp12-alkylating (red) and hydrolytic (teal) processes reveals the higher sensitivity of the latter to C3 substituents. **b**, Chemical structures of C3-substituted malolactones (*R*)-G12Di-2 (**2**), (2*R*,3*S*)-G12Di-3 (**3**), and (2*R*,3*S*)-G12Di-4 (**4**). **c**, Recombinant K-Ras-G12D (200 nM) labeling kinetics with 3-substituted malolactones (10 µM) (*n* = 3, replicates are plotted as individual data points). **d**, Stability of 3-substituted β-lactones in PBS (pH 7.4) by LC–MS (*n* = 3, replicates are plotted as individual data points).

Despite being a potent covalent ligand of recombinant K-Ras-G12D, (*RS*)-G12Di-1 was not stable in aqueous buffers at pH 7.4, precluding its use in cellular assays. We reasoned that its stability could be improved by attaching substituents at either or both sides of the α-carbon. Substitutions at the α-carbon will block the trajectory of incoming water nucleophiles from at least one side of the β-lactone ring (Fig. 2a, lower path). The same modification of the electrophile was predicted to have little impact on the S_N_2 attack by Asp12 because of the pseudo-staggered conformation of the lowest unoccupied molecular orbital of the C–O bond and the α-carbon steric hindrance (Fig. 2a, upper path).

To test this hypothesis, we prepared (2*R*,3*S*)-G12Di-3 (**3**) and (2*R*,3*S*)-G12Di-4 (**4**) with increasing steric hinderance at the pro-*S* position of the α-carbon (C_3_ in Fig. 2b). G12Di-2–4 maintained the ability to react with K-Ras-G12D, although the bulkier compounds exhibited decreased reaction rates (Fig. 2c). Meanwhile, (2*R*,3*S*)-G12Di-4, bearing an isopropyl group, resisted hydrolysis, with >90% of the material remaining intact after 24 h at 23 °C and pH 7.4. By contrast, (*R*)-G12Di-2 and (2*R*,3*S*)-G12Di-3 exhibited half-lives of 1.7 h and 2.5 h, respectively (Fig. 2d).

**Fig. 3:**
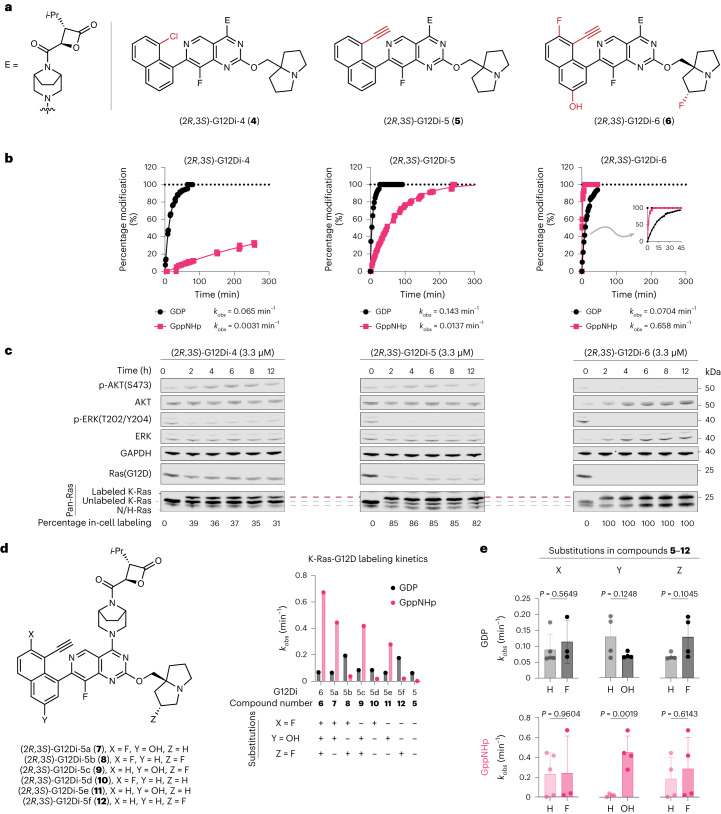
Rapid covalent modification of K-Ras-G12D•GTP is essential for in-cell target engagement and oncogenic signaling suppression. **a**, Chemical structures of (2*R*,3*S*)-G12Di-4 (**4**), (2*R*,3*S*)-G12Di-5 (**5**) and (2*R*,3*S*)-G12Di-6 (**6**). **b**, Recombinant K-Ras-G12D (200 nM) labeling kinetics with 3-isopropyl malolactones (10 µM) (*n* = 3, replicates are plotted as individual data points). **c**, Western blot time course of cellular K-Ras-G12D covalent engagement and downstream signaling inhibition. Data are representative of two independent experiments. **d**, Covalent K-Ras-G12D labeling kinetics in both nucleotides by (2*R*,3*S*)-G12Di-5, (2*R*,3*S*)-G12Di-6 and (2*R*,3*S*)-G12Di-5a–5f (**7**–**12**). Substitutions X, Y and Z vary between compounds **7**–**12**. **e**, Analysis of the determining substitution on covalent labeling kinetics by an unpaired *t*-test comparing X = H (*n* = 5) or F (*n* = 3), Y = H (*n* = 4) or OH (*n* = 4), and Z = H (*n* = 4) or F (*n* = 4). All data points represent individual chemical compound. Data are presented as mean ± standard deviation. Source data

With an improved isopropyl (*i*-Pr)-substituted malolactone electrophile, we further explored S-IIP ligands with higher affinity, including the 8-ethynylnaphthyl ligand in (*RS*)-G12Di-1 ((2*R*,3*S*)-G12Di-5 (**5**)) and MRTX1133 ((2*R*,3*S*)-G12Di-6 (**6**))^35–37^. The fast kinetics of GDP-state labeling persisted, (2*R*,3*S*)-G12Di-5 and (2*R*,3*S*)-G12Di-6 labeled the GTP-state up to 200-fold faster than (2*R*,3*S*)-G12Di-4, possibly due to the accessibility of the S-IIP of K-Ras-G12D•GTP to the MRTX1133 scaffold. When tested at 10 µM, compound (2*R*,3*S*)-G12Di-6 fully labeled 200 nM (ref. ^47^) of K-Ras-G12D•GppNHp within 5 min, which is, to our knowledge, the first S-IIP covalent molecule that has a preference for GTP state of K-Ras (Fig. 3b). Unlike MRTX1133, which prefers the GDP state^48^, its (2*R*,3*S*)-3-isopropyl malolactone derivative (2*R*,3*S*)-G12Di-6 preferred the GTP state as assessed by intact protein MS. The in-cell K-Ras-G12D covalent labeling kinetics using a western blot time course mirrored the recombinant protein results (Fig. 3c), where (2*R*,3*S*)-G12Di-6 labeled endogenous K-Ras-G12D completely in homozygous *KRAS*^G12D/G12D^ cancer cell line SW1990 within 2 h as indicated by the gel mobility shift in the anti-Ras immunoblot, and concomitant reduction of the phospho-ERK levels. This is consistent with the higher biochemical potency we observed with K-Ras-G12D•GppNHp, as cellular K-Ras-G12D is enriched in the GTP-bound state as a result of its poor intrinsic and GAP mediated GTPase activity^45^.

To understand the significantly improved GTP-state labeling kinetics from (2*R*,3*S*)-G12Di-5 to (2*R*,3*S*)-G12Di-6, we synthesized six compounds ((2*R*, 3*S*)-G12Di-5a (**7**) to (2*R*,3*S*)-G12Di-5f (**12**), Fig. 3d) exploiting their difference in the S-IIP binding core substitution pattern. Measuring the pseudo-first-order kinetic rates with 200 nM K-Ras-G12D and 10 µM compounds, GTP-state labeling was more sensitive to these substitution changes with higher coefficient of variation (CV_GppNHp_ 1.1, CV_GDP_ 0.5). We grouped compounds with or without certain substitution, and analyzed the means of pseudo-first-order rates using Student’s *t*-test (Fig. 3e). While the two fluorine substitutions on the naphthyl ring or the bicyclic tertiary amine were dispensable, naphthyl 3-OH substitution was critical for fast GTP-state covalent labeling (*P* = 0.0019). Besides the gain of GTP-state labeling kinetics, the 3-hydroxy in the naphthyl ring significantly stabilized the covalent complex by 5–9 °C (ΔΔ*T*_m_, 3-hydroxy group versus 3-hydrogen, Supplementary Table 6). We reason that the hydrogen bonding (2.7 Å, as measured in Protein Data Bank (PDB) access code 7T47) between naphthyl 3-OH and Asp69 side chain is critical for S-IIP inhibitors to access the GTP state and stabilize the complex. Similar interaction exists in previous GTP-state-selective cyclic peptide KD2 (ref. ^44^) and K-Ras-G12C inhibitors ARS-853 and ARS-107 (ref. ^34^), highlighting an underappreciated pharmacophore for active state targeting of K-Ras.

### Optimized ligand inhibits tumor growth in vitro and in vivo

**Extended Data Fig. 5:**
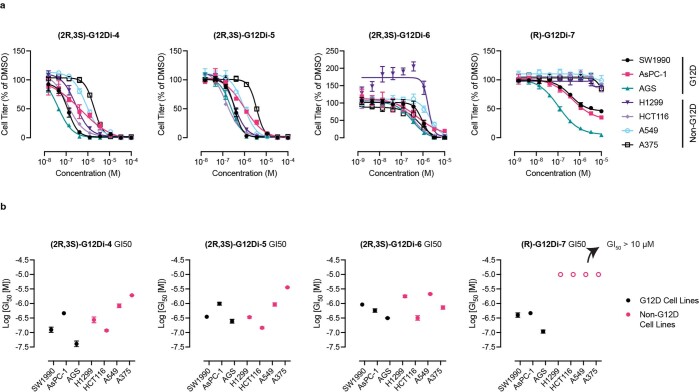
Cell growth inhibition of advanced β-lactone G12Di against a panel of G12D or non-G12D cell lines. **a**, Cell growth inhibition curves. All data points represent individual biological replicates. Data are presented as mean ± SD (*n* = 3). **b**, Half-maximal inhibitory concentrations (IC_50_s) of each biological replicate are plotted.

**Extended Data Fig. 6:**
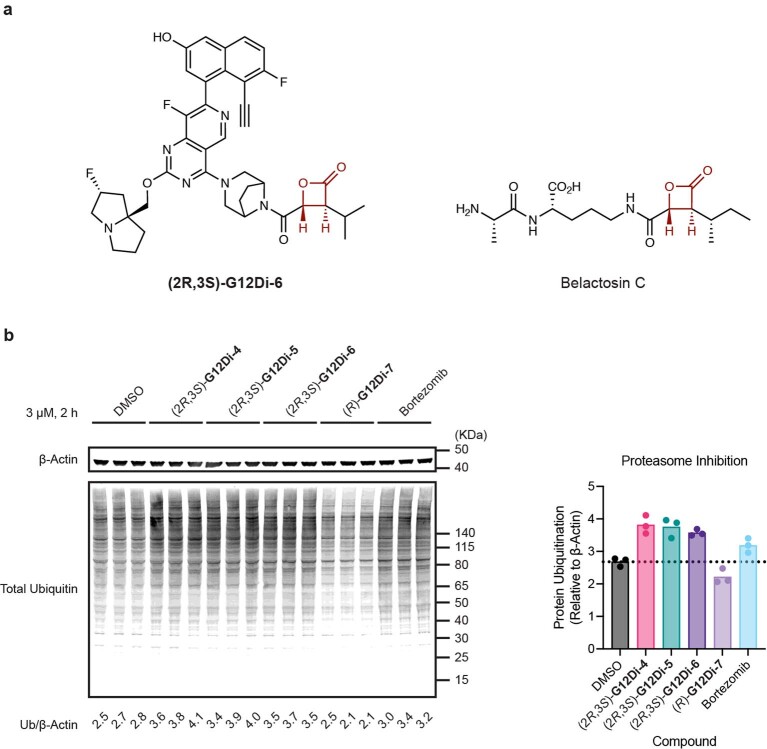
Malolactone compounds 4–6 inhibited 20 S proteasome while (*R*)-7 did not. **a**, Chemical structures of Compound 6 and Belactosin C with β-lactone warhead highlighted in red. **b**, Immunoblot of poly-ubiquitinated proteins in HEK293 cells All data points represent individual biological replicates. Data are presented as mean ± SD (*n* = 3). Source data

Despite efficient labeling of K-Ras-G12D by G12Di-4–6 in cells, these compounds did not show significant mutant selectivity in terms of cell growth inhibition in a survey of seven cancer cell lines including three *KRAS*^G12D^ mutant lines and four with either WT *KRAS* or non-G12D *KRAS* mutations (Extended Data Fig. 5a,b). We reasoned that G12Di-4–6 may possess cellular targets that are critical for cell survival other than K-Ras-G12D. We ruled out inhibition of WT K-Ras based on the effect on A375 cells that possess a downstream BRAF (V600E) activating mutation that should bypass K-Ras-WT inhibition. We next turned to the monosubstituted malolactone electrophile in G12Di-4–6, which resembles that found in the natural product belactosin C (refs. ^49–51^), a potent covalent inhibitor of the eukaryotic proteasome (Extended Data Fig. 6). We confirmed that compounds G12Di-4–6 inhibited proteasome activity and induced accumulation of poly-ubiquitinated proteins in HEK293 cells (Extended Data Fig. 6). Because proteasome inhibition leads to cell growth inhibition independent of K-Ras-G12D mutation, we sought to eliminate this off-target activity.

**Fig. 4:**
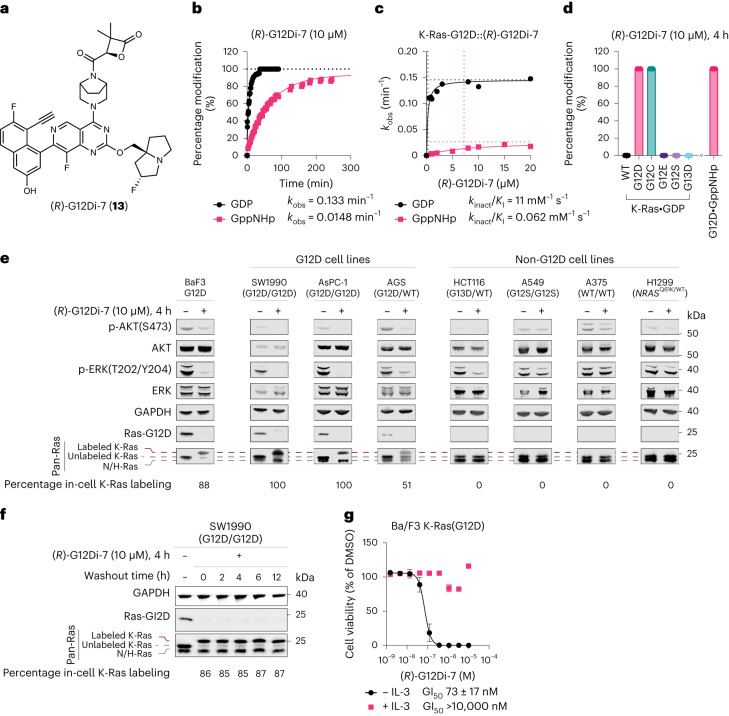
Malolactone (*R*)-G12Di-7 (13) covalently and mutant-selectively modified recombinant and endogenous K-Ras-G12D oncoprotein in cancer cell lines. **a**, Chemical structure of (*R*)-G12Di-7. **b**, Pseudo-first-order K-Ras-G12D labeling kinetics of (*R*)-G12Di-7. Conditions: K-Ras-G12D (200 nM), (*R*)-G12Di-7 (10 µM), room temperature. **c**, Second-order K-Ras-G12D labeling kinetics of (*R*)-G12Di-7. **d**, Covalent labeling selectivity against K-Ras WT and mutants. All data points represent individual biological replicates. Data are presented as mean ± standard deviation (*n* = 3). **e**, Immunoblot of Ba/F3:K-Ras-G12D, SW1990 AsPC-1, AGS, HCT116, A549, A375 and H1299 cells treated with DMSO or 10 µM (*R*)-G12Di-7 for 4 h. Data are representative of two independent experiments. **f**, Stability of covalent complex G12D•(*R*)-G12Di-7 in cells. Data are representative of two independent experiments. **g**, Relative growth of Ba/F3:K-Ras-G12D cells (with or without 10 ng ml^−1^ IL-3) after treatment with (*R*)-G12Di-7 for 72 h. Data are presented as mean ± standard deviation (*n* = 3) and are representative of two independent experiments. Source data

**Extended Data Fig. 7:**
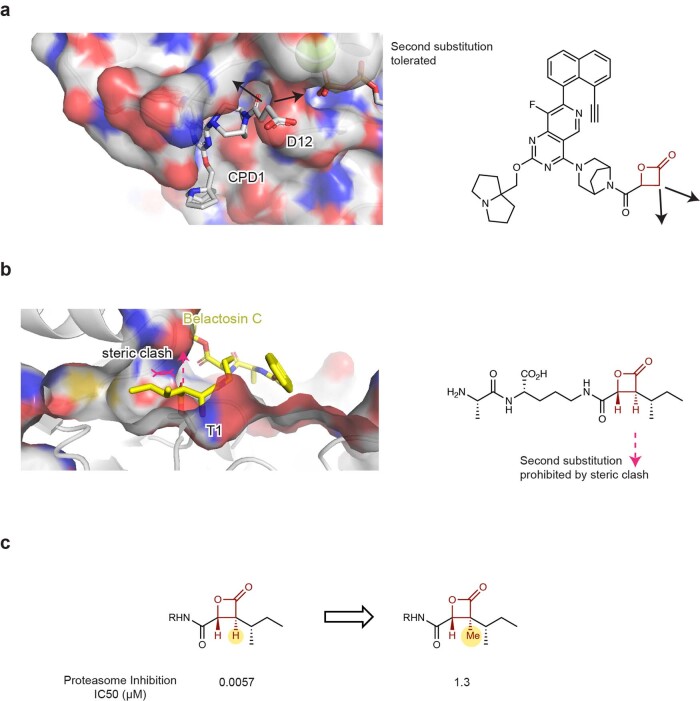
Analysis of X-ray co-crystal structures containing covalent malolactone electrophile. **a**, α-Carbon of malolactone G12Di could tolerate geminal disubstitution. **b**, Belactosin C could not tolerate a second α-substitution. **c**, Additional methyl group (circled in yellow) in belactosin analog caused significant loss of activity.

**Extended Data Fig. 8:**
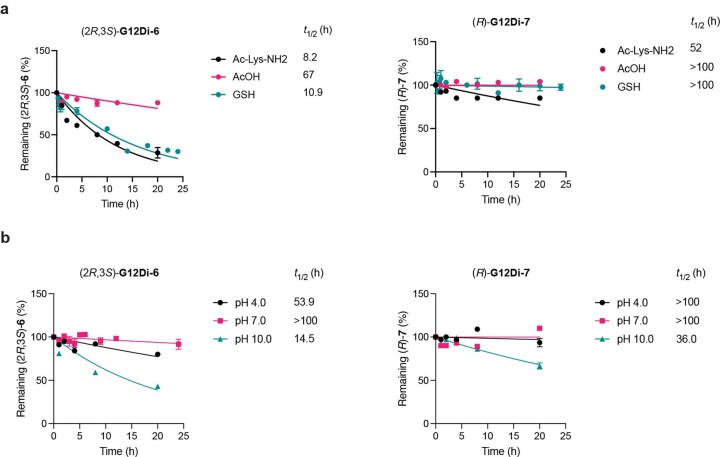
Intrinsic reactivity and stability of G12Di-6 and 7. **a**, Stability in the presence of excess compounds (5 mM) bearing reactive functional groups. All data points represent individual biological replicates. Data are presented as mean ± SD (*n* = 2). **b**, Stability in 1X PBS at various pH. All data points represent individual biological replicates. Data are presented as mean ± SD (*n* = 2).

**Extended Data Fig. 9:**
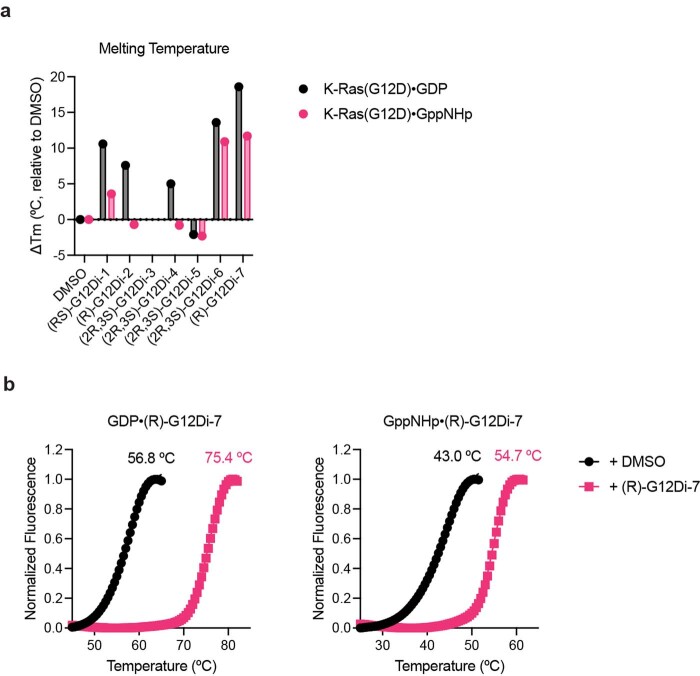
Melting point change of covalently crosslinked K-Ras-G12D. **a**, Summary of thermal stabilization of K-Ras-G12D by G12Di-1–7. **b**, DSF melting curves of K-Ras-G12D•GDP or GppNHp covalently bound to (*R*)-G12Di-7. All data points represent individual biological replicates. Data are presented as mean ± SD (*n* = 3).

Through comparison of the co-crystal structure of a belactosin C derivative bound to the yeast proteasome (PDB: 3TDD) and that of (*RS*)-G12Di-1 bound to K-Ras-G12D•GDP, we hypothesized that introduction of a second substitution at the pro-*R* position would block proteasome binding and further improve hydrolytic stability (Extended Data Fig. 7). Based on this design approach, we also reasoned that the larger pro-*S* isopropyl substitution introduced previously would not be required if both hydrogens were substituted and therefore designed a 3,3-*gem*-dimethyl substituted malolactone, (*R*)-G12Di-7 (**13**). As predicted, (*R*)-G12Di-7 with a doubly substituted electrophile showed excellent stability (*t*_1/2_ > 24 h) in the presence of reduced GSH in phosphate-buffered saline (PBS) at 37 °C for 24 h (Extended Data Fig. 8). (*R*)-G12Di-7 did not inhibit proteasome activity or induce protein poly-ubiquitination in HEK293 cells (Extended Data Fig. 6). We further show that (*R*)-G12Di-7 was highly selective for K-Ras-G12D and did not induce more than 20% inhibition in a panel of 482 kinases commonly used in safety profiling (Supplementary Table 10). (*R*)-G12Di-7 completely labeled K-Ras-G12D•GDP and K-Ras-G12D•GppNHp within 30 min and 300 min, respectively (Fig. 4b). The slower GppNHp-state labeling kinetics (cf. (2*R*,3*S*)-G12Di-6) was mainly due to the electrophile’s lower intrinsic reactivity (*k*_inact_) (Supplementary Table 5). (*R*)-G12Di-7 showed high mutant selectivity, labeling only the GDP and GppNHp state of G12D, but not the GDP state of WT, G12E, G12S or G13D despite elongated incubation time (Fig. 4d). The most reactive G12C mutant was also covalently labeled by (*R*)-G12Di-7. (*R*)-G12Di-7 stabilized both GDP and GppNHp-bound K-Ras-G12D by 18.6 °C and 11.7 °C, respectively (Extended Data Fig. 9).

The mutant-selective, in-cell covalent labeling was assessed by immunoblots, where (*R*)-G12Di-7 labeled endogenous K-Ras-G12D completely in homozygous *KRAS*^G12D/G12D^ cell lines SW1990 and AsPC-1, only half in the heterozygous *KRAS*^G12D/WT^ AGS cell line, and none in non-G12D mutation cell lines (Fig. 4e). The mutant selectivity was consistent between in-cell covalent modification and downstream signaling suppression. By contrast, the reversible inhibitor MRTX1133 is reported to inhibit WT KRAS signaling at concentrations >100 nM in mouse embryonic fibroblasts (MEF) cells engineered to express one single *RAS* allele (‘Rasless MEFs’)^38^. Washout experiment demonstrated the stability of the covalent K-Ras-G12D•(*R*)-G12Di-7 complex in cells over a course of 12 h (Fig. 4f).

To examine the on-target inhibition, we used *KRAS*^*G12D*^-transformed mouse pro-B cell line Ba/F3. The parental Ba/F3 cell line is dependent on interleukin 3 (IL-3) for survival and proliferation, while the transformed cells become independent of IL-3 but dependent on the transduced driver oncogene^52^. (*R*)-G12Di-7 inhibited growth of Ba/F3(*KRAS*^G12D^) cells with a GI_50_ of 73 ± 17 nM. The same cells co-treated with IL-3 (10 ng ml^−1^) lost sensitivity to (*R*)-G12Di-7 up to 10 µM suggesting the observed cell growth inhibition was due to on-target K-Ras-G12D inhibition (Fig. 4g).

**Fig. 5:**
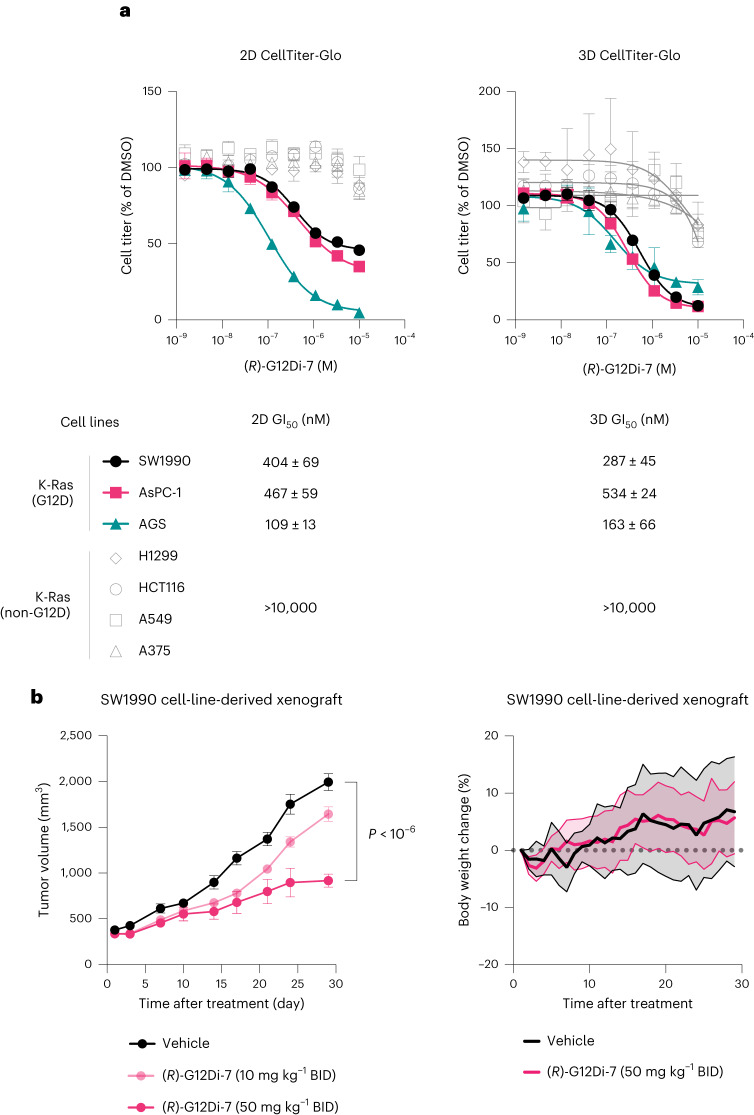
Covalent K-Ras-G12D inhibitor (*R*)-G12Di-7 selectively inhibits cell growth in cancer cell lines harboring *KRAS*^G12D^ mutation and tumor growth in mice bearing SW1990 xenograft. **a**, Relative growth of cancer cell lines with (black, red or teal) or without (gray) *KRAS*^G12D^ mutation after treatment with (*R*)-G12Di-7 for 72 h (2D) or 120 h (3D). Data are presented as mean ± standard deviation (*n* = 3) and are representative of two independent experiments. **b**, Tumor volumes and body weight of mice bearing SW1990 xenografts and treated with vehicle (10% captisol in 1× PBS, *n* = 8 biologically independent mice), (*R*)-G12Di-7 (10 mg kg^−1^, *n* = 9 biologically independent mice), or (*R*)-G12Di-7 (50 mg kg^−1^, *n* = 9 biologically independent mice). BID, twice a day. All data points represent individual biological replicates. Data are presented as mean ± standard error of the mean. Tumor volumes for the (*R*)-G12Di-7 (50 mg kg^−1^) treatment groups are statistically significant versus vehicle (*P* < 0.00001) by two-tailed Student’s *t*-test.

This compound further showed significantly biased inhibition profile toward K-Ras-G12D mutation (SW1990, AsPC-1 and AGS) from non-G12D mutation cancer cell lines (H1299, HCT116, A549 and A375) in 2D-adherent monolayer cultures. (*R*)-G12Di-7 showed minimal toxicity to the latter cell lines at concentrations up to 10 µM. Similar cell growth inhibition potency as well as selectivity was observed in 3D-spheroid suspensions^39^ (Fig. 5a). The cellular activity of (*R*)-G12Di-7 could be translated to a K-Ras-G12D cell line-derived xenograft in mice. We observed a dose-dependent inhibition of SW1990 xenograft growth as measured by volume upon treatment of (*R*)-G12Di-7 over a course of 28 days (Fig. 5b). No apparent toxicity induced by compound treatment was evidenced by the comparable weight gain over the treatment period in both groups of mice.

**Extended Data Fig. 10:**
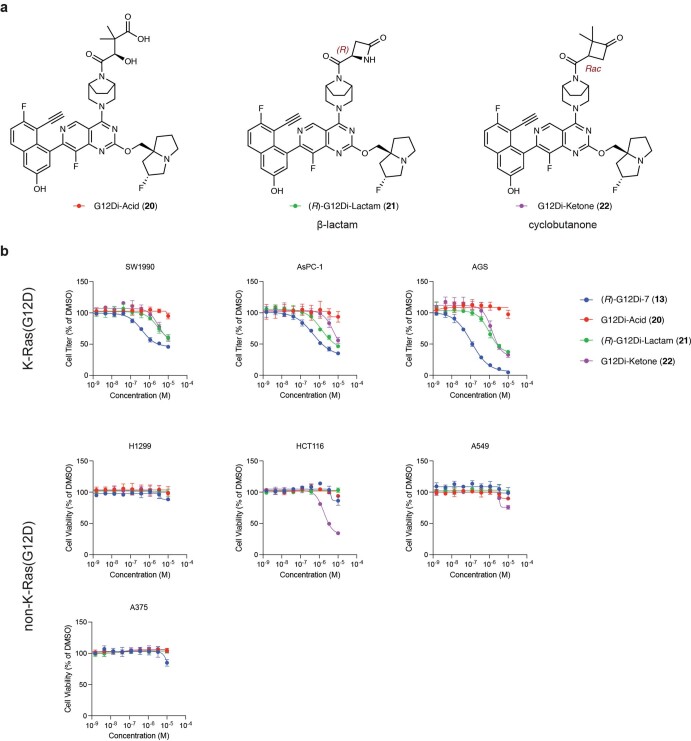
Covalency of malolactone is important for the G12D cell growth inhibition. **a**, Chemical structures of covalent K-Ras-G12D inhibitor and non-covalent analogs. **b**, Non-covalent analogs inhibited K-Ras-G12D cell lines with significantly lower potency. All data points represent individual biological replicates. Data are presented as mean ± SD (*n* = 3).

Further, we demonstrated the importance of covalency by testing three noncovalent analogs of our lead molecule (*R*)-G12Di-7, including its hydrolyzed product and two nonlactone cyclic analogs, β-lactam and cyclobutanone (Extended Data Fig. 10). The noncovalent analogs with amide pendants demonstrated moderate (>1 µM) GI_50_ values against K-Ras-G12D cell lines. Coupled with a covalent bond formation event in (*R*)-G12Di-7, the S-IIP engagement was significantly augmented leading to submicromolar GI_50_ values for K-Ras-G12D mutation cells and higher mutant selectivity. These results suggested that strong reversible S-IIP engagement, well-tuned G12D reactivity and hydrolytic stability are critical for achieving potent and selective cellular inhibition.

## Discussion

The discovery that the mutant cysteine in K-Ras-G12C can be exploited by small-molecule electrophiles has opened a new way to target oncogenic K-Ras-driven cancers. This has led to the recent US Food and Drug Administration approval of sotorasib and adagrasib. However, the much larger patient population bearing an acquired aspartic acid missense mutation at the same K-Ras Gly12 position remains a significant therapeutic challenge at the forefront of current research. This is partially due to the significantly lower nucleophilicity of aspartate compared to that of cysteine in aqueous solution^2,53,54^. The malolactone ligands described here efficiently covalently modify the mutant aspartate in K-Ras (G12D) in both the GDP- and GTP-bound states. The GDP/GTP dual state inhibitors hold the potential to provide desirable pharmacodynamics compared to inactive-state K-Ras inhibitors. One particular malolactone example ((2*R*,3*S*)-G12Di-6) predominantly labeled the GTP-bound state in K-Ras-G12D compared to the GDP state, leading to faster in-cell covalent modification and signaling pathway suppression (Fig. 3c). We further uncovered a critical hydrogen bonding interaction between S-IIP ligands and K-Ras Asp69 that contributes to the GTP-state engagement. It will be interesting to further explore the molecular determinants of GTP-state-preferring ligands especially in the context of pan-KRAS^4^ reversible ligands as this approach may provide some measure of WT-sparing activity since the GDP state predominates in the WT compared to the oncogenic mutants.

Although it is believed that the liable ester linkages are readily cleaved in the cytosolic environment^55^, time course immunoblots suggested that the K-Ras-G12D•malolactone complex remains intact for at least 12 h. Our observation suggests that ester bonds protected by the protein host in the ligand-binding pocket may be more resistant to intrinsic or esterase-mediated hydrolysis, a feature that could be exploited in the development of carboxylate-targeting covalent ligands beyond K-Ras-G12D inhibitors.

Together, our data demonstrate that strain release can be exploited to trap a common somatically mutated aspartate residue on the surface of the oncogene K-Ras-G12D. Compound (*R*)-G12Di-7 is a covalent ligand of K-Ras-G12D with potent and mutant-selective activity against K-Ras-G12D cancers in vitro and in vivo.

## Methods

### Ethics of animal use

All animal studies were performed at Crown Bioscience (San Diego, CA). All the animal study procedures were performed in the specific-pathogen-free animal facility at Crown Bioscience under the approved protocols by the Institutional Animal Care and Use Committee, with the guidance of the Association for Assessment and Accreditation of Laboratory Animal Care.

### Chemical synthesis and characterizations

Synthetic procedures and characterizations of new compounds are reported in Supplementary Information.

### Recombinant protein expression and purification

K-Ras-WT, K-Ras-G12D, K-Ras-G12E, K-Ras-G13D and K-Ras-G12D Cyslight DNA sequences encoding human K-Ras (WT, a.a. 1–169), human K-Ras (G12D, a.a. 1–169), human K-Ras (G12E, a.a. 1–169), human K-Ras (G13D, a.a. 1–169) and human K-Ras G12D Cyslight (G12D/C51S/C80L/C118S, a.a. 1–169) were codon optimized, synthesized by Twist Biosciences and cloned into pJExpress411 vector using the Gibson Assembly method^7^. The resulting construct contains N-terminal 6xHis tag and a Tobacco Etch Virus (TEV) protease cleavage site (ENLYFQG). The proteins were expressed and purified following previously reported protocols^2,8^. Briefly, chemically competent BL21(DE3) cells were transformed with the corresponding plasmid and grown on LB agar plates containing 50 µg ml^−1^ kanamycin. A single colony was used to inoculate a culture at 37 °C, 220 rpm in terrific broth containing 50 µg ml^−1^ kanamycin. When the optical density reached 0.6, the culture temperature was reduced to 20 °C, and protein expression was induced by the addition of isopropyl β-d-1-thiogalactopyranoside to 1 mM. After 16 h at 20 °C, the cells were pelleted by centrifugation (6,500*g*, 10 min) and lysed in lysis buffer (20 mM Tris 8.0, 500 mM NaCl and 5 mM imidazole) with a high-pressure homogenizer (Microfluidics). The lysate was clarified by high-speed centrifugation (19,000*g*, 15 min) and the supernatant was used in subsequent purification by immobilized metal affinity chromatography. His-TEV-tagged protein was captured with Co-TALON resin (Clonetech, Takara Bio, 2 ml slurry per liter culture) at 4 °C for 1 h with constant end-to-end mixing. The loaded beads were then washed with lysis buffer (50 ml per liter culture), and the protein was eluted with elution buffer (20 mM Tris 8.0, 300 mM NaCl and 300 mM imidazole). To this protein solution was added His-tagged TEV protease (0.05 mg TEV per milligram Ras protein) and GDP (1 mg per milligram Ras protein), and the mixture was dialyzed against TEV cleavage buffer (20 mM Tris 8.0, 300 mM NaCl, 1 mM ethylenediaminetetraacetic acid and 1 mM DTT) at 4 °C using a 10 K molecular weight cutoff (MWCO) dialysis cassette until liquid chromatography (LC)–MS analysis showed full cleavage (typically 16–24 h). MgCl_2_ was added to a final concentration of 5 mM, and the mixture was incubated with 1 ml Ni-NTA (Qiagen) beads at 4 °C for 1 h to remove TEV protease, any residual His-tagged proteins and peptides. The protein solution was diluted 1:10 v/v with 20 mM Tris 8.0 and further purified with anion exchange chromatography (HiTrapQ column, GE Healthcare Life Sciences) using a NaCl gradient of 50 mM to 500 mM in 20 mM Tris 8.0. Nucleotide loading was performed by mixing the ion exchange-purified protein with an excess of GDP (5 mg per liter culture) or GppNHp (5 mg per liter culture) and 5 mM ethylenediaminetetraacetic acid at 23 °C for 30 min. The reaction was stopped by the addition of MgCl_2_ to 10 mM. For GppNHp, an additional calf intestine phosphatase treatment was performed as follows to ensure high homogeneity of the loaded nucleotide. The protein buffer was exchanged into phosphatase buffer (32 mM Tris 8.0, 200 mM ammonium sulfate and 0.1 mM ZnCl_2_) with a HiTrap Desalting Column (GE Healthcare Life Sciences). To the buffer-exchanged protein solutions, GppNHp was added to 5 mg ml^−1^, and calf intestine phosphatase (NEB) was added to 10 U ml^−1^. The reaction mixture was incubated on ice for 1 h, and MgCl_2_ was added to a final concentration of 20 mM. After nucleotide loading, the protein was concentrated using an 10 K MWCO centrifugal concentrator (Amicon-15, Millipore) to 20 mg ml^−1^ and purified by size exclusion chromatography on a Superdex 75 10/300 GL column (GE Healthcare Life Sciences). Fractions containing pure Ras protein were pooled and concentrated to 20 mg ml^−1^ and stored at −80 °C. In our hands, this protocol gives a typical yield of 5–15 mg per liter culture.

### Crystallization

K-Ras-G12D Cyslight (G12D/C51S/C80L/C118S) bound by GDP purified by size exclusion chromatography was diluted to 20 µM in reaction buffer (20 mM HEPES 7.5, 150 mM NaCl and 1 mM MgCl_2_). Compound **1** was added as a 10 mM solution in dimethyl sulfoxide (DMSO) to a final concentration of 50 µM. The mixture was allowed to stand at 23 °C until LC–MS analysis of the reaction mixture showed full conversion to a single covalent adduct. The reaction mixture was concentrated using a 10 K MWCO filter device and the adduct was purified by size exclusion chromatography (Superdex 75, 20 mM HEPES 7.5, 150 mM NaCl and 1 mM MgCl_2_) and concentrated to 20 mg ml^−1^. For crystallization, 0.1 µl of the protein was mixed with 0.1 µl well buffer containing 0.1 M MES 6.5, 25% PEG4K. Crystals were grown at 20 °C in a 96-well plate using the hanging-drop vapor diffusion method. Maximal crystal growth was achieved after 7 days. The crystals were transferred to a cryoprotectant solution (0.1 M MES 6.5, 25% PEG4K and 25% glycerol) and flash-frozen in liquid nitrogen.

### X-ray data collection and structure determination

The dataset was collected at the Advanced Light Source beamline 8.2.2 with X-ray at a wavelength of 0.999907 Å. The dataset was indexed and integrated using iMosflm^56^, scaled with Scala^57^ and solved by molecular replacement using Phaser^58^ in CCP4 software suite^59^. The crystal structure of GDP-bound K-Ras-G12C-MRTX849 adduct (PDB code 6USZ) was used as the initial model. The structure was manually refined with Coot^60^ and PHENIX^61^. Data collection and refinement statistics are listed in Supplementary Table 1. In the Ramachandran plot of the final structure, 98.21% and 1.49% of the residues are in the favored regions and allowed regions, respectively.

### Cell culture

AsPc-1 (CRL-1682), SW1990 (CRL-2172), H1299 (or NCI-H1299, CRL-5803), HCT-116 (CCL-247), A549 (CRM-CCL-185), A375 (or A-375, CRL-1619), HEK293 (CRL-1573) cells were obtained from ATCC and maintained in high-glucose (4.5 g l^−1^) DMEM (Gibco 11995073) supplemented with 4 mM l-glutamine, 1 mM sodium pyruvate and 10% heat-inactivated fetal bovine serum (FBS; Axenia Biologix). AGS (CRL-1739) cells were obtained from ATCC and maintained in Ham’s F12-K (Gibco 21127022) supplemented with 10% heat-inactivated FBS (Axenia Biologix). Ba/F3 cells were a gift from Dr. Trever Bivona (University of California, San Francisco) and were maintained in RPMI-1640 (Gibco 11875093) supplemented with 10% heat-inactivated FBS (Axenia Biologix) and 10 ng ml^−1^ recombinant mouse IL-3 (Gibco PMC0031).

Cells were passed for at least two generations after cryorecovery before they were used for assays. All cell lines were tested mycoplasma negative using MycoAlert Mycoplasma Detection Kit (Lonza).

### Gel electrophoresis and immunoblotting

Cells were treated with drugs at 40–60% confluency at a final DMSO concentration of 1%. At the end of the treatment period, cells were chilled on ice. Unless otherwise indicated, adherent cells were washed once with ice-cold PBS (1 ml), scraped with a spatula, and pelleted by centrifugation (500*g*, 5 min). Suspension cells were pelleted by centrifugation (500*g*, 5 min), washed with 1 ml ice-cold PBS, and pelleted again. Cells were lysed in RIPA buffer supplemented with protease and phosphatase inhibitors (mini cOmplete and phosSTOP, Roche) on ice for 10 min. Concentrations of lysates were determined with protein BCA assay (Thermo Fisher) and adjusted to 2 mg ml^−1^ or lowest available concentration with additional RIPA buffer. Samples were mixed with 5× sodium dodecyl sulfate (SDS) loading dye and denatured at either room temperature for 30 min for Ras band shift experiment, or 95 °C for 5 min for other non-Ras proteins immunoblotting. Note: protein denaturation with 5× SDS loading dye at elevated temperature caused hydrolysis of covalent K-Ras-G12D•malolactone adducts due to the action of DTT. The same covalent complex remained stable at room temperature with 500 mM DTT.

Unless otherwise noted, SDS–polyacrylamide gel electrophoresis was run with Novex 12% Bis-Tris gel (Invitrogen) in MOPS running buffer (Invitrogen) at 200 V for 60 min following the manufacturer’s instructions. Proteins were transferred onto 0.2-µm nitrocellulose membranes (Bio-Rad) using a wet-tank transfer apparatus (Bio-Rad Criterion Blotter) in 1× TOWBIN buffer with 10% methanol at 75 V for 45 min. Membranes were blocked in 5% bovine serum albumin (BSA)–tris-buffered saline-Tween 20 (TBST) for 1 h at 23 °C. Primary antibody binding was performed with the indicated antibodies diluted in 5% BSA–TBST at 4 °C for at least 16 h. After washing the membrane three times with TBST (5 min each wash), secondary antibodies (goat anti-rabbit IgG-IRDye 800 and goat anti-mouse IgG-IRDye 680, Li-COR) were added as solutions in 5% BSA–TBST at the dilutions recommended by the manufacturer. Secondary antibody binding was allowed to proceed for 1 h at 23 °C. The membrane was washed three times with TBST (5 min each wash) and imaged on a Li-COR Odyssey fluorescence imager.

### Preparation of MSCV

pMSCV-Puro plasmids (where MSCV is mouse stem cell virus) containing full-length human *KRAS* genes (WT, G12D) were constructed using standard molecule biology techniques by inserting the *KRAS* gene fragment between the BamHI and XhoI sites. Transfection-grade plasmids were prepared using ZymoPure II Plasmid Midiprep kit. EcoPack 293 cells (Takara Bio) were plated in six-well plates (3 × 10^5^ ml^−1^, 2 ml). The next day, cells were transfected with 2.5 µg pMSCV plasmid using Lipofectamine 3000 following the manufacturer’s instructions. The cells were incubated for 66 h, and then the virus-containing supernatants were collected and passed through a 0.22-µm syringe filter. The collected virus was used immediately for spinfection of Ba/F3 cells or stored at −80 °C.

### Generation of stable Ba/F3 transductants

One milliliter of MSCV-containing supernatant (vide supra) was added to one well of a six-well plate containing 1 × 10^6^ Ba/F3 cells in 1 ml of medium composed of 60% RMPI 1640, 40% heat-inactivated fetal bovine serum (FBS), 10 ng mouse IL-3 and 4 µg polybrene. Cells were spinfected by centrifugation at 2,000*g* for 90 min at room temperature and then placed in the incubator for 24 h. After 1 day, the cells were diluted into 10 ml culture medium (RPMI 1640 + 10% heat-inactivated FBS, 10 ng ml^−1^ mouse IL-3) and recovered for a second day after spinfection. On the third day after spinfection, cells were pelleted at 500*g* for 5 min and resuspended in 10 ml selection medium (RPMI 1640 + 10% heat-inactivated FBS, 10 ng ml^−1^ mouse IL-3 and 1.25 µg ml^−1^ puromycin). Cells were maintained under puromycin selection for 4–7 days, splitting as required to maintain density <2 × 10^6^ cells ml^−1^. After 7 days, cells were pelleted, washed once with IL-3 free culture medium (RPMI 1640 + 10% heat-inactivated FBS) and pelleted again before resuspending at 2–4 × 10^5^ cells ml^−1^ in IL-3 free culture medium. Cells were maintained under these conditions for 7 days, passaging as needed to maintain density <2 × 10^6^ cells ml^−1^. Growth was monitored (Countess II Cell Counter) over these 7 days to confirm that an IL-3 independent population has been achieved.

### Differential scanning fluorimetry

The protein of interest was diluted with SEC buffer (20 mM HEPES 7.5, 150 mM NaCl and 1 mM MgCl_2_) to 2 µM. This solution was dispensed into wells of a white 96-well polymerase chain reaction plate in triplicate (25 µl per well). Fluorescence was measured at 0.5 °C temperature intervals every 30 s from 25 °C to 95 °C on a Bio-Rad CFX96 qPCR system using the FRET setting. Each dataset was normalized to the highest fluorescence and the normalized fluorescence reading was plotted against temperature in GraphPad Prism 9.0. *T*_m_ values were determined as the temperature(s) corresponding to the maximum (or maxima) of the first derivative of the curve. Proteins crosslinked with small molecules were desalted using Zeba Spin Desalting Columns (Thermo) before differential scanning fluorimetry *T*_m_ measurement.

### Detection of covalent modification of K-Ras by whole-protein MS

Test compounds were prepared as 100× stock solutions in DMSO. K-Ras proteins were diluted with SEC buffer (20 mM HEPES 7.5, 150 mM NaCl and 1 mM MgCl_2_) to 1 µM. In a typical reaction, 1 µl 100× compound stock was mixed with 99 µl diluted K-Ras protein, and the resulting mixture was incubated for the desired amount of time. The extent of modification was assessed by electrospray MS using a Waters Xevo G2-XS system equipped with an Acquity UPLC BEH C4 1.7 µm column. The mobile phase was a linear gradient of 5–95% acetonitrile/water + 0.05% formic acid. For kinetic measurements, a 2× compound solution was first prepared in SEC buffer, which was then mixed with 400 nM K-Ras-G12D protein at 1:1 (v/v) ratio. Injection time stamps were used to calculate elapsed time.

### Detection of covalent modification of K-Ras by tandem MS

K-Ras-G12D protein (1 µM, 100 µl) in PBS 7.4 was treated with β-propiolactone (1 mM or 10 mM) at 23 °C for 1 h. The reaction buffer was exchanged into digestion buffer (20 mM Tris 8.0 and 2 mM CaCl_2_) using a Zeba 0.5-ml desalting column (7 K MWCO, Thermo Scientific). Eighty microliters of the resulting protein solution was mixed with 2 µl 200 mM DTT. The mixture was heated at 56 °C for 30 min. After cooling to 23 °C, 4 µl 200 mM iodoacetamide was added. After 15 min at 23 °C, 2.1 µl 200 mM DTT was added. After an additional 5 min, 500 ng trypsin was added to the mixture, and the samples were incubated at 37 °C overnight. Five microliters 10% formic acid was added to stop the digestion (final formic acid concentration 0.5% v/v). The tryptic peptides were enriched and desalted using OMIX C18 tips (Agilent) following the manufacturer’s instructions. Peptides (0.5% of total) were resolved on an Easy-Spray nano-HPLC column (Thermo Fisher ES800A, 150 mm length, 3 µl particle size, 100-Å particle size) over a 54-min gradient of 2–37% acetonitrile–water + 0.1% formic acid and analyzed by a Q-Exactive hybrid quadrupole-Orbitrap mass spectrometer (MS1 resolution: 70,000; automatic gain control (AGC) target, 3 × 10^6^; range, 350–1,500 *m*/*z*; MS2 resolution: 17,500; AGC target, 5 × 10^4^; maximum injection time, 120 ms; Top 10: NCE, 25%; dynamic exclusion, 15 s). Peptides were searched against the K-Ras-G12D sequence using MaxQuant (v.2.0.3.0, https://www.maxquant.org/), with β-propiolactone (C_3_H_4_O_2_) as a variable modification on serine, threonine, lysine, aspartate, tyrosine, glutamate and histidine residues. Peptides were identified with a false discovery rate cutoff of 0.01. Only the peptides with sufficient MS2 fragment information to assign the modification site with >0.9 probability were used for analysis.

### Stability of β-lactone compounds in PBS

Ten-micromolar solution of β-lactone compound in PBS, pH 7.4 was prepared by diluting 1 µl of 10 mM DMSO stock solution into 999 µl of PBS pH 7.4 in the presence or absence of 5 mM reduced GSH. At specified time points, aliquots were taken, and the amount of intact compound was analyzed on a Waters Xevo G2-XS Quadrupole-TOF system equipped with an Acquity UPLC BEH C18 1.7 µm column for multiple-reaction monitoring for the 1+ precursor of respective compound (precursor > precursor) with target enhancement and low collision energy (0–2 eV).

### 2D cell viability assay

Cells were seeded into 96-well white flat-bottom plates (1,000 cells per well) (Greiner Bio-One, 655083) and incubated overnight. Cells were treated with the indicated compounds in a nine-point threefold dilution series (100 μl final volume) and incubated for 72 h. Cell viability was assessed using a commercial CellTiter-Glo (CTG) luminescence-based assay (Promega). The 96-well plates were equilibrated to room temperature before the addition of diluted CTG reagent (100 μl) (1:4 CTG reagent:PBS, containing 1% Triton X-100). Plates were placed on an orbital shaker for 30 min before recording luminescence using a Spark 20M (Tecan) plate reader.

### 3D cell viability assay

Cells were resuspended in fresh culture medium to a concentration of (1,000 cells per well) and plated (90 µl per well) in Corning Costar 3474, 96-well clear flat-bottom ultralow-attachment microplates. Cells were treated with the indicated compounds in a nine-point threefold dilution series (100 μl final volume) and incubated for 120 h. Cell viability was assessed using a commercial CTG luminescence-based assay (Promega) as described in 2D cell viability assay.

### Animal studies

All animal studies were performed at Crown Bioscience (San Diego, CA). Six- to 9-week-old female mice (NOD/SCID) were purchased from the Jackson Laboratory. SW1990 xenografts were established by subcutaneous injection into the rear flanks of mice with SW1990 cells (5 × 10^6^ cells in 100 μl of serum-free medium mixed 1:1 with Matrigel). Tumor xenografts were allowed to establish, and mice were randomized into control and treatment groups when tumors reached a size range of 200–400 mm^3^. Dosing of (*R*)-G12Di-7 (10 or 50 mg kg^−1^ in 5% DMSO, 10% captisol in 1× PBS pH 7.4) or vehicle control (5% DMSO, 10% captisol in 1× PBS pH 7.4) was administered twice a day via intraperitoneal route. Tumor volume was assessed biweekly and body weight was assessed daily for the duration of the study. Tumor volume was assessed by caliper 2D measurement, and volume was calculated using the following formula to approximate the volume: (longest diameter × shortest diameter^2^)/2.

### Reporting summary

Further information on research design is available in the Nature Portfolio Reporting Summary linked to this article.

## Online content

Any methods, additional references, Nature Portfolio reporting summaries, source data, extended data, supplementary information, acknowledgements, peer review information; details of author contributions and competing interests; and statements of data and code availability are available at 10.1038/s41589-024-01565-w.

### Supplementary information

Supplementary Information Supplementary Figs. 1 and 2, Tables 1–10, chemical synthesis and characterizations, References 62–67 and nuclear magnetic resonance spectra.

Reporting Summary

### Source data

Source Data Fig. 3 Uncropped western blot images for Fig. 3c.

Source Data Fig. 4 Uncropped western blot images for Fig. 4e.

Source Data Fig. 4 Uncropped western blot images for Fig. 4e (continued).

Source Data Fig. 4 Uncropped western blot images for Fig. 4f.

Source Data Extended Data Fig. 6 Uncropped western blot images for Extended Data Fig. 6.

## Data Availability

Atomic coordinates and structure factors for the reported crystal structure have been deposited with the PDB, with the following accession numbers: K-Ras-G12D•GDP•(*RS*)-G12Di-1, 8T4V. Additional data used in this study are PDB 7PRZ, 6UT0 and 6OIM. Protein sequences used in this study could be accessed at Uniprot (https://uniprot.org) with the following accession code: P01116 (K-Ras, the 1–169 truncated form was used). Source data are provided with this paper.

## References

[1] Prior, I. A., Hood, F. E. & Hartley, J. L. The frequency of Ras mutations in cancer. *Cancer Res.* **80**, 2969–2974 (2020). 32209560 10.1158/0008-5472.CAN-19-3682 PMC7367715

[2] Ostrem, J. M., Peters, U., Sos, M. L., Wells, J. A. & Shokat, K. M. K-Ras(G12C) inhibitors allosterically control GTP affinity and effector interactions. *Nature* **503**, 548–551 (2013). 24256730 10.1038/nature12796 PMC4274051

[3] Hallin, J. et al. Anti-tumor efficacy of a potent and selective non-covalent KRASG12D inhibitor. *Nat. Med.* **28**, 2171–2182 (2022). 36216931 10.1038/s41591-022-02007-7

[4] Kim, D. et al. Pan-KRAS inhibitor disables oncogenic signalling and tumour growth. *Nature*10.1038/s41586-023-06123-3 (2023). 37258666 10.1038/s41586-023-06123-3 PMC10322706

[5] Ma, N. et al. 2*H*-azirine-based reagents for chemoselective bioconjugation at carboxyl residues inside live cells. *J. Am. Chem. Soc.* **142**, 6051–6059 (2020). 32159959 10.1021/jacs.9b12116

[6] McGrath, N. A., Andersen, K. A., Davis, A. K. F., Lomax, J. E. & Raines, R. T. Diazo compounds for the bioreversible esterification of proteins. *Chem. Sci.* **6**, 752–755 (2015). 25544883 10.1039/C4SC01768D PMC4275067

[7] Jun, J. V., Petri, Y. D., Erickson, L. W. & Raines, R. T. Modular diazo compound for the bioreversible late-stage modification of proteins. *J. Am. Chem. Soc.* **145**, 6615–6621 (2023). 36920197 10.1021/jacs.2c11325 PMC10175043

[8] Knox, J. E. et al. RM-036, a first-in-class, orally-bioavailable, Tri-Complex covalent KRASG12D(ON) inhibitor, drives profound anti-tumor activity in KRASG12D mutant tumor models. in *American Association for Cancer Research (AACR) Annual Meeting* (2022).

[9] Zanon, P. R. A. et al. Profiling the proteome-wide selectivity of diverse electrophiles. Preprint at *ChemRxiv*10.26434/chemrxiv.14186561.v1 (2021). 10.1038/s41557-025-01902-z PMC12580327 41168333

[10] Abbasov, M. E. et al. A proteome-wide atlas of lysine-reactive chemistry. *Nat. Chem.* **13**, 1081–1092 (2021). 34504315 10.1038/s41557-021-00765-4 PMC8952960

[11] Yang, T. et al. Reversible lysine-targeted probes reveal residence time-based kinase selectivity. *Nat. Chem. Biol.* **18**, 934–941 (2022). 35590003 10.1038/s41589-022-01019-1 PMC9970282

[12] Wan, X. et al. Discovery of lysine-targeted eIF4E inhibitors through covalent docking. *J. Am. Chem. Soc.* **142**, 4960–4964 (2020). 32105459 10.1021/jacs.9b10377 PMC7136196

[13] Chen, W. et al. Arylfluorosulfates inactivate intracellular lipid binding protein(s) through chemoselective SuFEx reaction with a binding site Tyr residue. *J. Am. Chem. Soc.* **138**, 7353–7364 (2016). 27191344 10.1021/jacs.6b02960 PMC4909538

[14] Zhang, Z., Guiley, K. Z. & Shokat, K. M. Chemical acylation of an acquired serine suppresses oncogenic signaling of K-Ras(G12S). *Nat. Chem. Biol.* **18**, 1177–1183 (2022). 35864332 10.1038/s41589-022-01065-9 PMC9596369

[15] Zheng, Q. et al. SuFEx-enabled, agnostic discovery of covalent inhibitors of human neutrophil elastase. *Proc. Natl Acad. Sci. USA* **116**, 18808–18814 (2019). 31484779 10.1073/pnas.1909972116 PMC6754619

[16] Zhang, Z., Morstein, J., Ecker, A. K., Guiley, K. Z. & Shokat, K. M. Chemoselective covalent modification of K-Ras(G12R) with a small molecule electrophile. *J. Am. Chem. Soc.* **144**, 15916–15921 (2022). 36001446 10.1021/jacs.2c05377 PMC9460778

[17] Gonzalez-Valero, A. et al. An activity-based oxaziridine platform for identifying and developing covalent ligands for functional allosteric methionine sites: redox-dependent inhibition of cyclin-dependent kinase 4. *J. Am. Chem. Soc.* **144**, 22890–22901 (2022). 36484997 10.1021/jacs.2c04039 PMC10124963

[18] Lowther, W. T., McMillen, D. A., Orville, A. M. & Matthews, B. W. The anti-angiogenic agent fumagillin covalently modifies a conserved active-site histidine in the *Escherichia coli* methionine aminopeptidase. *Proc. Natl Acad. Sci. USA* **95**, 12153–12157 (1998). 9770455 10.1073/pnas.95.21.12153 PMC22800

[19] Jia, S., He, D. & Chang, C. J. Bioinspired thiophosphorodichloridate reagents for chemoselective histidine bioconjugation. *J. Am. Chem. Soc.* **141**, 7294–7301 (2019). 31017395 10.1021/jacs.8b11912 PMC6996876

[20] Cruite, J. T. et al. Cereblon covalent modulation through structure-based design of histidine targeting chemical probes. *RSC Chem. Biol.* **3**, 1105–1110 (2022). 36128501 10.1039/D2CB00078D PMC9428674

[21] McGregor, L. M., Jenkins, M. L., Kerwin, C., Burke, J. E. & Shokat, K. M. Expanding the scope of electrophiles capable of targeting K-Ras oncogenes. *Biochemistry* **56**, 3178–3183 (2017). 28621541 10.1021/acs.biochem.7b00271 PMC5665167

[22] Wang, H.-L., Cee, V. J., Parsons, A. T. & Beaver, M. Pyridopyrimidine derivatives useful as KRAS G12C and KRAS G12D inhibitors in the treatment of cancer. WO 2021/081212 A1 (2021).

[23] Yu, Z. et al. Simultaneous covalent modification of K-Ras(G12D) and K-Ras(G12C) with tunable oxirane electrophiles. *J. Am. Chem. Soc.* **145**, 20403–20411 (2023). 37534597 10.1021/jacs.3c05899

[24] Robinson, S. L., Christenson, J. K. & Wackett, L. P. Biosynthesis and chemical diversity of β-lactone natural products. *Nat. Prod. Rep.* **36**, 458–475 (2019). 30191940 10.1039/C8NP00052B

[25] Hassan, A. Q. et al. The novolactone natural product disrupts the allosteric regulation of Hsp70. *Chem. Biol.* **22**, 87–97 (2015). 25544045 10.1016/j.chembiol.2014.11.007

[26] She, Y. M., Cheng, K. D., Farnsworth, A., Li, X. G. & Cyr, T. D. Surface modifications of influenza proteins upon virus inactivation by beta-propiolactone. *Proteomics* **13**, 3537–3547 (2013). 24123778 10.1002/pmic.201300096 PMC4265195

[27] Logrippo, G. A. Investigations of the use of beta-propiolactone in virus inactivation. *Ann. N. Y. Acad. Sci.* **83**, 578–594 (1960). 14417982 10.1111/j.1749-6632.1960.tb40931.x

[28] Budowsky, E. I., Friedman, E. A., Zheleznova, N. V. & Noskov, F. S. Principles of selective inactivation of viral genome. VI. Inactivation of the infectivity of the influenza virus by the action of beta-propiolactone. *Vaccine* **9**, 398–402 (1991). 1887669 10.1016/0264-410X(91)90125-P

[29] Fan, C. et al. Beta-propiolactone inactivation of coxsackievirus A16 induces structural alteration and surface modification of viral capsids. *J. Virol.* **91**, 10-1128 (2017). 10.1128/JVI.00038-17 PMC5375664 28148783

[30] Gao, Q. et al. Development of an inactivated vaccine candidate for SARS-CoV-2. *Science* **369**, 77–81 (2020). 32376603 10.1126/science.abc1932 PMC7202686

[31] Wang, H. et al. Development of an inactivated vaccine candidate, BBIBP-CorV, with potent protection against SARS-CoV-2. *Cell* **182**, 713–721 (2020). 32778225 10.1016/j.cell.2020.06.008 PMC7275151

[32] Böttcher, T. & Sieber, S. A. β-Lactams and β-lactones as activity-based probes in chemical biology. *MedChemComm* **3**, 408–417 (2012). 10.1039/c2md00275b

[33] Zhang, Y., Gross, R. A. & Lenz, R. W. Stereochemistry of the ring-opening polymerization of (*S*)-β-butyrolactone. *Macromolecules* **23**, 3206–3212 (1990). 10.1021/ma00215a002

[34] Hansen, R. et al. The reactivity-driven biochemical mechanism of covalent KRAS(G12C) inhibitors. *Nat. Struct. Mol. Biol.* **25**, 454–462 (2018). 29760531 10.1038/s41594-018-0061-5

[35] Wang, X. et al. KRAS G12D inhibitors. WO/2021/041671 (2021).

[36] Vasta, J. D. et al. KRAS is vulnerable to reversible Switch-II Pocket engagement in cells. *Nat. Chem. Biol.* **18**, 596–604 (2022). 35314814 10.1038/s41589-022-00985-w PMC9135634

[37] Wang, X. et al. Identification of MRTX1133, a noncovalent, potent, and selective KRAS(G12D) inhibitor. *J. Med. Chem.* **65**, 3123–3133 (2022). 34889605 10.1021/acs.jmedchem.1c01688

[38] Keats, M. A., Han, J. J. W., Lee, Y.-H., Lee, C.-S. & Luo, J. A nonconserved histidine residue on KRAS drives paralog selectivity of the KRASG12D inhibitor MRTX1133. *Cancer Res.* **83**, 2816–2823 (2023). 37339170 10.1158/0008-5472.CAN-23-0592 PMC10528543

[39] Janes, M. R. et al. Targeting KRAS mutant cancers with a covalent G12C-specific inhibitor. *Cell* **172**, 578–589 (2018). 29373830 10.1016/j.cell.2018.01.006

[40] Lanman, B. A. et al. Discovery of a covalent inhibitor of KRAS(G12C) (AMG 510) for the treatment of solid tumors. *J. Med. Chem.* **63**, 52–65 (2020). 31820981 10.1021/acs.jmedchem.9b01180

[41] Fell, J. B. et al. Identification of the clinical development candidate MRTX849, a covalent KRAS(G12C) inhibitor for the treatment of cancer. *J. Med. Chem.* **63**, 6679–6693 (2020). 32250617 10.1021/acs.jmedchem.9b02052

[42] Lorthiois, E. et al. JDQ443, a structurally novel, pyrazole-based, covalent inhibitor of KRASG12C for the treatment of solid tumors. *J. Med. Chem.* **65**, 16173–16203 (2022). 36399068 10.1021/acs.jmedchem.2c01438

[43] Schulze, C. J. et al. Chemical remodeling of a cellular chaperone to target the active state of mutant KRAS. *Science* **381**, 794–799 (2023). 37590355 10.1126/science.adg9652 PMC10474815

[44] Zhang, Z. et al. GTP-state-selective cyclic peptide ligands of K-Ras(G12D) block its interaction with Raf. *ACS Cent. Sci.* **6**, 1753–1761 (2020). 33145412 10.1021/acscentsci.0c00514 PMC7596874

[45] Hunter, J. C. et al. Biochemical and structural analysis of common cancer-associated KRAS mutations. *Mol. Cancer Res* **13**, 1325–1335 (2015). 26037647 10.1158/1541-7786.MCR-15-0203

[46] Olson, A. R. & Miller, R. J. The mechanism of the aqueous hydrolysis of β-butyrolactone. *J. Am. Chem. Soc.* **60**, 2687–2692 (1938). 10.1021/ja01278a041

[47] Fujioka, A. et al. Dynamics of the Ras/ERK MAPK cascade as monitored by fluorescent probes. *J. Biol. Chem.* **281**, 8917–8926 (2006). 16418172 10.1074/jbc.M509344200

[48] Peacock, D. M., Kelly, M. J. S. & Shokat, K. M. Probing the KRas Switch II groove by fluorine NMR spectroscopy. *ACS Chem. Biol.* **17**, 2710–2715 (2022). 36166818 10.1021/acschembio.2c00566 PMC9594042

[49] Asai, A., Hasegawa, A., Ochiai, K., Yamashita, Y. & Mizukami, T. Belactosin A, a novel antitumor antibiotic acting on cyclin/CDK mediated cell cycle regulation, produced by *Streptomyces* sp. *J. Antibiot.* **53**, 81–83 (2000). 10.7164/antibiotics.53.81 10724015

[50] Kawamura, S. et al. Potent proteasome inhibitors derived from the unnatural cis-cyclopropane isomer of belactosin A: synthesis, biological activity, and mode of action. *J. Med. Chem.* **56**, 3689–3700 (2013). 23547757 10.1021/jm4002296

[51] Kawamura, S., Unno, Y., Asai, A., Arisawa, M. & Shuto, S. Design and synthesis of the stabilized analogs of belactosin A with the unnatural cis-cyclopropane structure. *Org. Biomol. Chem.* **11**, 6615–6622 (2013). 23986389 10.1039/c3ob41338a

[52] Warmuth, M., Kim, S., Gu, X.-J., Xia, G. & Adrián, F. Ba/F3 cells and their use in kinase drug discovery. *Curr. Opin. Oncol.* **19**, 55–60 (2007). 17133113 10.1097/CCO.0b013e328011a25f

[53] Mayr, H. & Patz, M. Scales of nucleophilicity and electrophilicity: a system for ordering polar organic and organometallic reactions. *Angew. Chem. Int. Ed. Engl.* **33**, 938–957 (1994). 10.1002/anie.199409381

[54] Lito, P., Solomon, M., Li, L.-S., Hansen, R. & Rosen, N. Allele-specific inhibitors inactivate mutant KRAS G12C by a trapping mechanism. *Science* **351**, 604–608 (2016). 26841430 10.1126/science.aad6204 PMC4955282

[55] Mix, K. A., Lomax, J. E. & Raines, R. T. Cytosolic delivery of proteins by bioreversible esterification. *J. Am. Chem. Soc.* **139**, 14396–14398 (2017). 28976737 10.1021/jacs.7b06597 PMC5856659

[56] Battye, T. G., Kontogiannis, L., Johnson, O., Powell, H. R. & Leslie, A. G. iMOSFLM: a new graphical interface for diffraction-image processing with MOSFLM. *Acta Crystallogr. D* **67**, 271–281 (2011). 21460445 10.1107/S0907444910048675 PMC3069742

[57] Evans, P. Scaling and assessment of data quality. *Acta Crystallogr. D* **62**, 72–82 (2006). 16369096 10.1107/S0907444905036693

[58] McCoy, A. J. et al. Phaser crystallographic software. *J. Appl. Crystallogr.* **40**, 658–674 (2007). 19461840 10.1107/S0021889807021206 PMC2483472

[59] Winn, M. D. et al. Overview of the CCP4 suite and current developments. *Acta Crystallogr. D* **67**, 235–242 (2011). 21460441 10.1107/S0907444910045749 PMC3069738

[60] Emsley, P., Lohkamp, B., Scott, W. G. & Cowtan, K. Features and development of Coot. *Acta Crystallogr. D* **66**, 486–501 (2010). 20383002 10.1107/S0907444910007493 PMC2852313

[61] Adams, P. D. et al. PHENIX: a comprehensive Python-based system for macromolecular structure solution. *Acta Crystallogr. D* **66**, 213–221 (2010). 20124702 10.1107/S0907444909052925 PMC2815670

