# Supplementary material for: Strain-release alkylation of Asp12 enables mutant selective targeting of K-Ras-G12D
Source: Nat Chem Biol. 2024 Mar 5;20(9):1114–22. doi: 10.1038/s41589-024-01565-w (PMC11357986; doi:10.1038/s41589-024-01565-w)
Supplement: Supplementary file 2 — Reporting Summary [file 41589_2024_1565_MOESM2_ESM.pdf]

Corresponding author(s): Shokat, Kevan M.
Zhang, Ziyang

Last updated by author(s): Jan 6, 2024

Reporting Summary

Nature Portfolio wishes to improve the reproducibility of the work that we publish. This form provides structure for consistency and transparency in reporting. For further information on Nature Portfolio policies, see our Editorial Policies and the Editorial Policy Checklist.

Statistics

For all statistical analyses, confirm that the following items are present in the figure legend, table legend, main text, or Methods section.

n/a	Confirmed	
☐	☒	The exact sample size (*n*) for each experimental group/condition, given as a discrete number and unit of measurement
☐	☒	A statement on whether measurements were taken from distinct samples or whether the same sample was measured repeatedly
☐	☒	The statistical test(s) used AND whether they are one- or two-sided<br>*Only common tests should be described solely by name; describe more complex techniques in the Methods section.*
☒	☐	A description of all covariates tested
☒	☐	A description of any assumptions or corrections, such as tests of normality and adjustment for multiple comparisons
☐	☒	A full description of the statistical parameters including central tendency (e.g. means) or other basic estimates (e.g. regression coefficient) AND variation (e.g. standard deviation) or associated estimates of uncertainty (e.g. confidence intervals)
☐	☒	For null hypothesis testing, the test statistic (e.g. F, t, r) with confidence intervals, effect sizes, degrees of freedom and P value noted<br>*Give P values as exact values whenever suitable.*
☒	☐	For Bayesian analysis, information on the choice of priors and Markov chain Monte Carlo settings
☒	☐	For hierarchical and complex designs, identification of the appropriate level for tests and full reporting of outcomes
☒	☐	Estimates of effect sizes (e.g. Cohen's *d*, Pearson's *r*), indicating how they were calculated

Our web collection on statistics for biologists contains articles on many of the points above.

Software and code

Policy information about availability of computer code

Data collection	Western Blot: LICOR Odyssey 2.1<br>Plate Reader: TECAN SparkControl 2.1<br>LC-MS: Waters MassLynx 4.2
Data analysis	GraphPad Prism 10.0.2<br>MestReNova 14.2.0<br>ImageJ 1.53k<br>CCP4i2 1.0.2 (including iMosflm 7.4.0, Aimless 0.7.4, and Phaser 2.8.3 modules)<br>Coot 0.9.6<br>Phenix 1.19.2<br>MaxQuant 2.0.3.1

For manuscripts utilizing custom algorithms or software that are central to the research but not yet described in published literature, software must be made available to editors and reviewers. We strongly encourage code deposition in a community repository (e.g. GitHub). See the Nature Portfolio guidelines for submitting code & software for further information.

Data

Policy information about availability of data

All manuscripts must include a data availability statement. This statement should provide the following information, where applicable:

- Accession codes, unique identifiers, or web links for publicly available datasets
- A description of any restrictions on data availability
- For clinical datasets or third party data, please ensure that the statement adheres to our policy

Atomic coordinates and structure factors for the reported crystal structures have been deposited with the Protein Data Bank (PDB), with the following accession numbers: K-Ras(G12D)•GDP•1, 8T4V.
Additional data used in this study are PDB 7PRZ, 6UT0, 6OIM.
Protein sequences used in this study are at Uniprot (https://uniprot.org) with the following accession codes P01116 (K-Ras, the 1-169 short form was used).
Source data of uncropped, unprocessed gel images are provide with this paper.

Research involving human participants, their data, or biological material

Policy information about studies with human participants or human data. See also policy information about sex, gender (identity/presentation), and sexual orientation and race, ethnicity and racism.

Reporting on sex and gender	N/A
Reporting on race, ethnicity, or other socially relevant groupings	N/A
Population characteristics	N/A
Recruitment	N/A
Ethics oversight	N/A

Note that full information on the approval of the study protocol must also be provided in the manuscript.

Field-specific reporting

Please select the one below that is the best fit for your research. If you are not sure, read the appropriate sections before making your selection.

☒ Life sciences ☐ Behavioural & social sciences ☐ Ecological, evolutionary & environmental sciences

For a reference copy of the document with all sections, see nature.com/documents/nr-reporting-summary-flat.pdf

Life sciences study design

All studies must disclose on these points even when the disclosure is negative.

Sample size	For plate reader based assays, three technical replicates were performed in each experiment. All experiments except crystallography have been performed in at least two biological replicates. See figure legends for exact number of biological replicates for each experiment.<br>For mouse experiment, at least eight mice were used for each group. The determination of the sample size was based on achieving the minimum number of animals required for sacrifice while also ensuring statistical validity. The exact sample size was indicated in the figure legend.
Data exclusions	No data were excluded from analysis.
Replication	All attempts for replication were successful (see figure legends for the number of replicates for each experiment).<br>We did not replicate X-ray crystallography experiment because of its nature.
Randomization	All mice xenografted with tumor were randomized in assigning to treatment and control groups.<br>Besides mouse experiments, each biochemical experiment in this study is rationally designed and leads to a specific conclusion. Thus, samples were not randomized.
Blinding	Samples were not blinded. Each biochemical experiment in this study is rationally designed and leads to a specific conclusion.

Reporting for specific materials, systems and methods

We require information from authors about some types of materials, experimental systems and methods used in many studies. Here, indicate whether each material, system or method listed is relevant to your study. If you are not sure if a list item applies to your research, read the appropriate section before selecting a response.

Materials & experimental systems

n/a	Involved in the study
☐	☒ Antibodies
☐	☒ Eukaryotic cell lines
☒	☐ Palaeontology and archaeology
☐	☒ Animals and other organisms
☒	☐ Clinical data
☒	☐ Dual use research of concern
☒	☐ Plants

Methods

n/a	Involved in the study
☒	☐ ChIP-seq
☒	☐ Flow cytometry
☒	☐ MRI-based neuroimaging

Antibodies

Antibodies used

Phospho-Akt (Ser473), Cell Signaling Technology, #4060, D9E
Akt, Cell Signaling Technology, #2920, 40D4
Phospho-p44/42 MAPK (Erk1/2) (Thr202/Tyr204), Cell Signaling Technology, #9101, Polyclonal
p44/42 MAPK (Erk1/2), Cell Signaling Technology, #4695, 137F5
Ras (G12D Mutant), Invitrogen, #MA5-36256, HL10
Ras, Abcam, #ab108602, EPR3255
GAPDH, ProteinTech, #60004-1-lg, 1E6D9
Ubiquitin, Santa Cruz Biotechnology, #sc-8017, P4D1
β-Actin, Cell Signaling Technology, #8457, D6A8
goat anti-rabbit IgG-IRDye 800, LI-COR, 926-32211, Clone # Not Available
goat anti-mouse IgG-IRDye 680, LI-COR, 926-68070, Clone # Not Available

Validation

All commercial antibodies have been validated by the manufacturers, see below:
Phospho-Akt (Ser473), https://www.cellsignal.com/products/primary-antibodies/phospho-akt-ser473-d9e-xp-rabbit-mab/4060
Akt, https://www.cellsignal.com/products/primary-antibodies/akt-pan-40d4-mouse-mab/2920
Phospho-p44/42 MAPK (Erk1/2) (Thr202/Tyr204), https://www.cellsignal.com/products/primary-antibodies/phospho-p44-42-mapk-erk1-2-thr202-tyr204-antibody/9101
p44/42 MAPK (Erk1/2), https://www.cellsignal.com/products/primary-antibodies/p44-42-mapk-erk1-2-137f5-rabbit-mab/4695
Ras (G12D Mutant), https://www.thermofisher.com/antibody/product/Ras-G12D-Mutant-Antibody-clone-HL10-Monoclonal/MA5-36256
Ras, https://www.abcam.com/products/primary-antibodies/ras-antibody-epr3255-ab108602.html
GAPDH, https://www.ptglab.com/products/GAPDH-Antibody-60004-1-Ig.htm
Ubiquitin, https://www.scbt.com/p/ubiquitin-antibody-p4d1
β-Actin, https://www.cellsignal.com/products/primary-antibodies/b-actin-d6a8-rabbit-mab/8457
goat anti-rabbit IgG-IRDye 800, https://www.licor.com/bio/reagents/irdye-800cw-goat-anti-rabbit-igg-secondary-antibody?utm_source=google&utm_medium=adwords&utm_content=reagent-webpage&utm_campaign=reagents&gclid=Cj0KCQjwgNanBhDUARIsAAeIcAvn3g3GbfE8Gir0ag7cn1ZFmFJXBKe__ip5ndT2hgRNNmCQV4IVSmcaAo6yEALw_wcB
goat anti-mouse IgG-IRDye 680, https://www.licor.com/bio/reagents/irdye-680rd-goat-anti-mouse-igg-secondary-antibody?utm_source=google&utm_medium=adwords&utm_content=reagent-webpage&utm_campaign=reagents&gclid=Cj0KCQjwgNanBhDUARIsAAeIcAt6VaxQsaaWaLsaFCTUplk4_RDD4Cy9KkbAJkn_wsSRPv7jAIky5m0aAr05EALw_wcB

Eukaryotic cell lines

Policy information about cell lines and Sex and Gender in Research

Cell line source(s)

SW1990: American Type Culture Collection (ATCC)
AsPC-1: American Type Culture Collection (ATCC)
AGS: American Type Culture Collection (ATCC)
H1299: American Type Culture Collection (ATCC)
HCT116: American Type Culture Collection (ATCC)
A549: American Type Culture Collection (ATCC)
A375: American Type Culture Collection (ATCC)
Ba/F3: German Collection of Microorganisms and Cell Cultures GmbH (DSMZ)
HEK293: American Type Culture Collection (ATCC)

Authentication

Cell lines from ATCC and DSMZ were STR profiled by the manufacturer.

Mycoplasma contamination

All cell lines were tested mycoplasma negative using MycoAlert TM Mycoplasma Detection Kit (Lonza).

Commonly misidentified lines
(See ICLAC register)

None

Animals and other research organisms

Policy information about studies involving animals; ARRIVE guidelines recommended for reporting animal research, and Sex and Gender in Research

Laboratory animals	6-9 week old, female, NOD/SCID mice. All animals were housed for a minimum 6-day stabilization period. Animals were observed daily for any clinical signs of disease. Animals were housed in individual HEPA ventilated cages (Innocage® IVC, Innovive USA). Fluorescent lighting was provided on a 12-hour cycle. Temperature and humidity were monitored and recorded daily and maintained to the maximum extent possible between 68-79°F (20-26°C) and 30-70% humidity, respectively. 2920X.10 18% soy irradiated rodent feed (Envigo) was provided and available ad libitum. Autoclaved acidified water (pH 2.5-3) was supplied ad libitum to all animals. Cage changes were performed every 2 weeks.
Wild animals	No wild animals were used.
Reporting on sex	Only female mice were used in these studies. Sex was not considered in study design in these experiments as the studies focus on cancer signaling.
Field-collected samples	Study did not involve samples collected from the field.
Ethics oversight	All animal studies were performed at Crown Bioscience (San Diego, CA). All the animal study procedures were performed in the SPF animal facility at Crown Bioscience under the approved protocols by the IACUC, with the guidance of the Association for Assessment and Accreditation of Laboratory Animal Care.

Note that full information on the approval of the study protocol must also be provided in the manuscript.

Plants

Seed stocks	N/A
Novel plant genotypes	N/A
Authentication	N/A
