# Supplementary figures and images for: Strain-release alkylation of Asp12 enables mutant selective targeting of K-Ras-G12D
Source: Nat Chem Biol. 2024 Mar 5;20(9):1114–22. doi: 10.1038/s41589-024-01565-w (PMC11357986; doi:10.1038/s41589-024-01565-w)

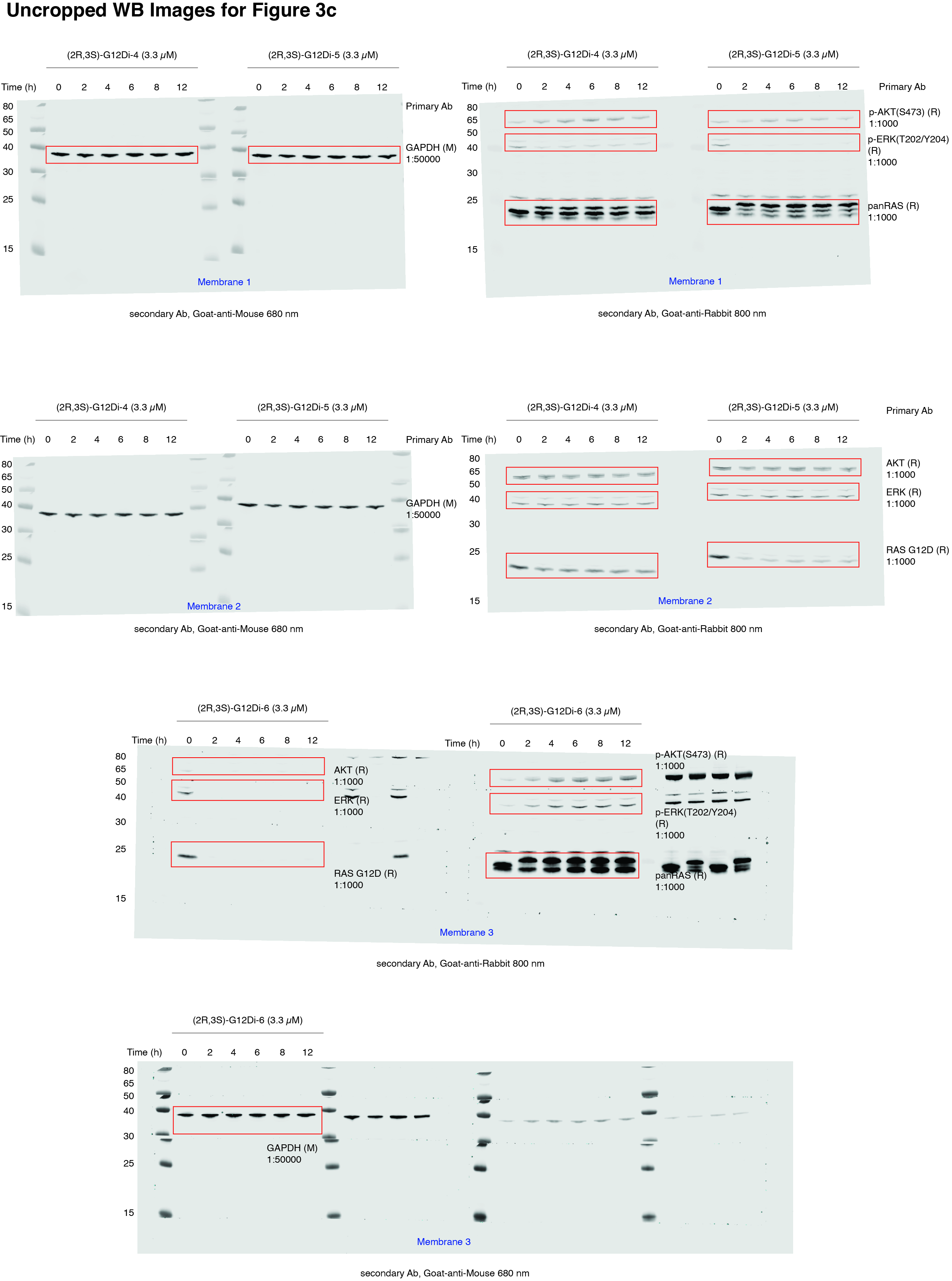

Supplement: Supplementary file 3 — Uncropped western blot images for Fig. 3c. [file 41589_2024_1565_MOESM3_ESM.jpg]

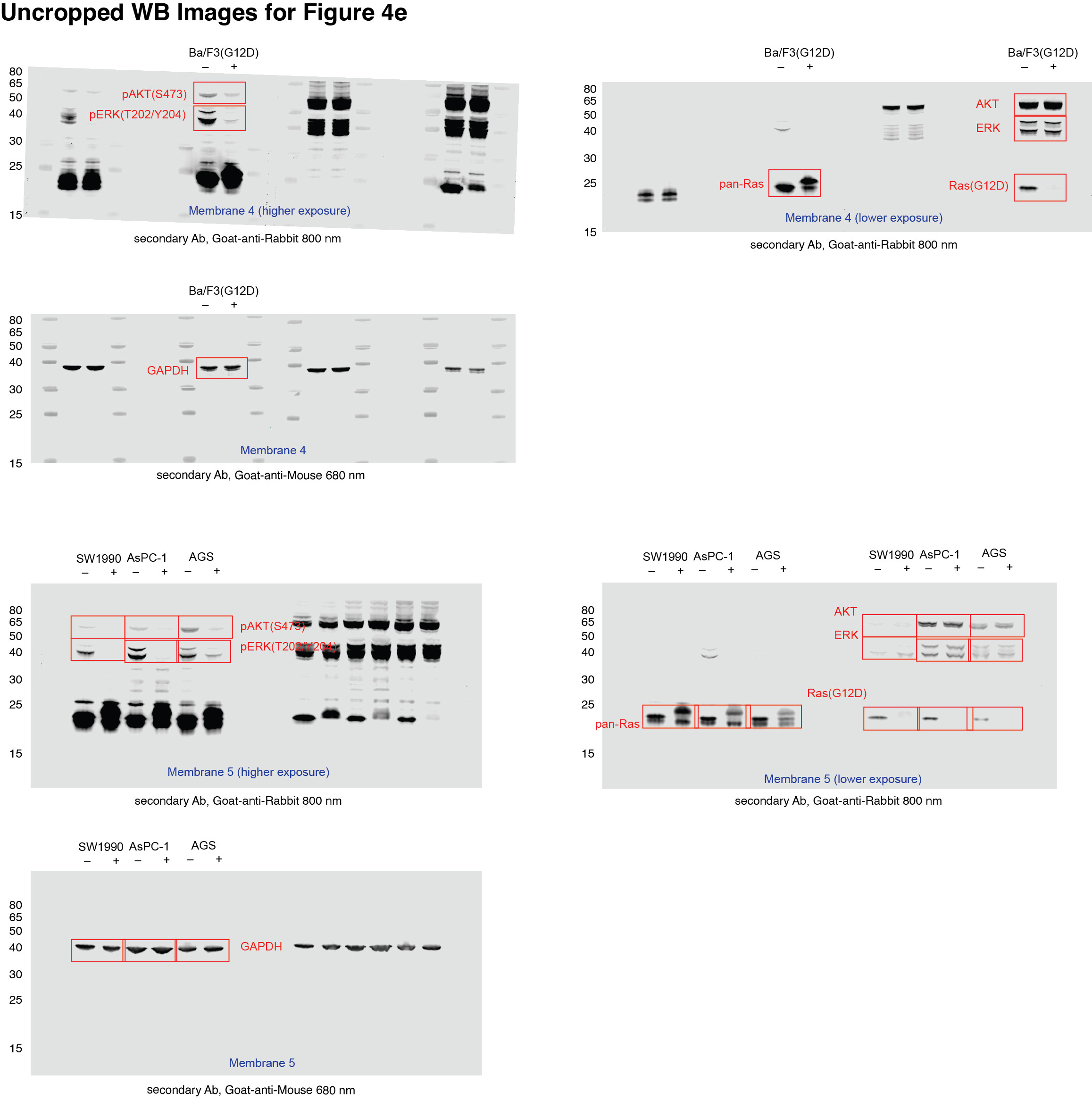

Supplement: Supplementary file 4 — Uncropped western blot images for Fig. 4e. [file 41589_2024_1565_MOESM4_ESM.jpg]

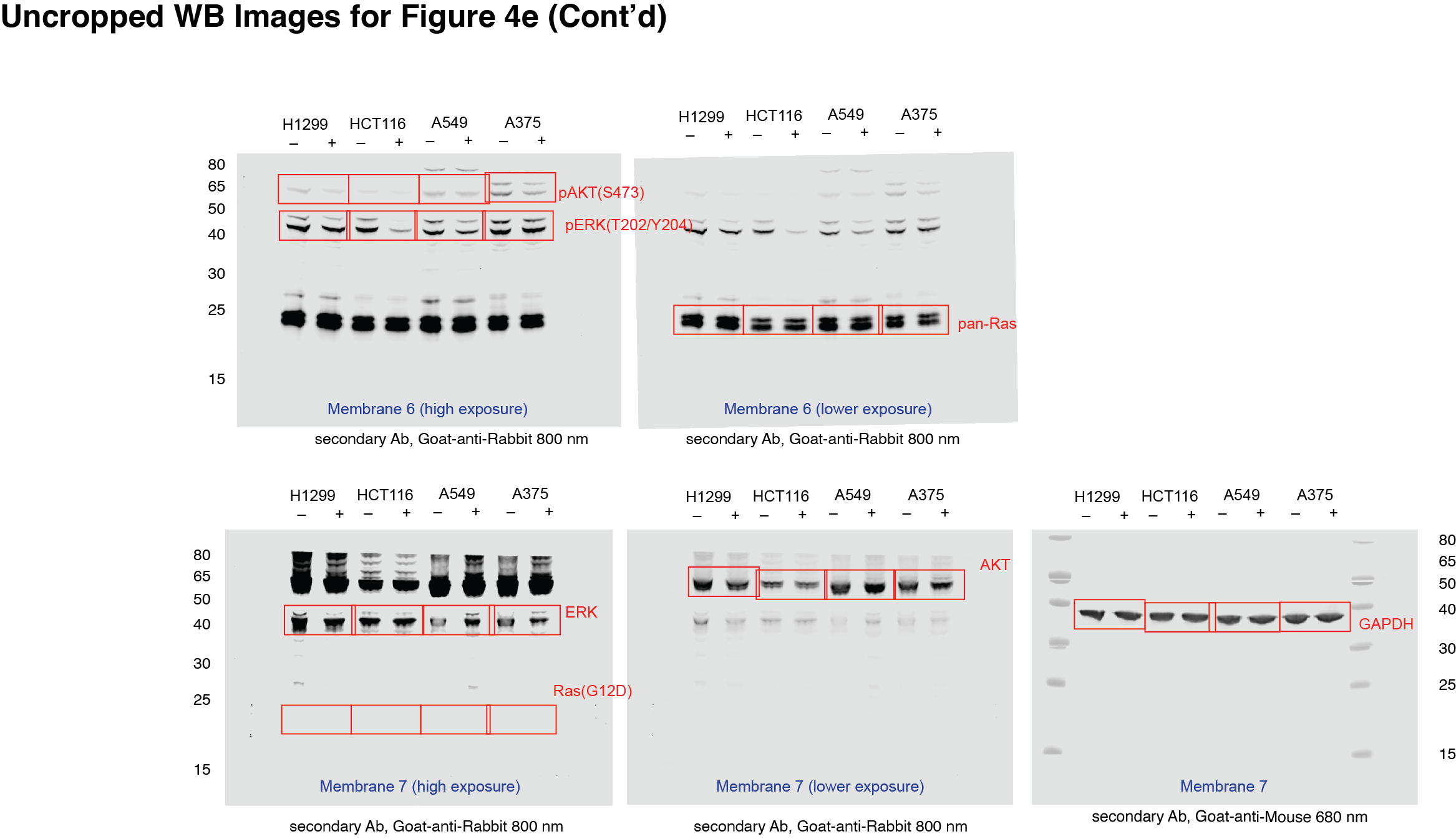

Supplement: Supplementary file 5 — Uncropped western blot images for Fig. 4e (continued). [file 41589_2024_1565_MOESM5_ESM.jpg]

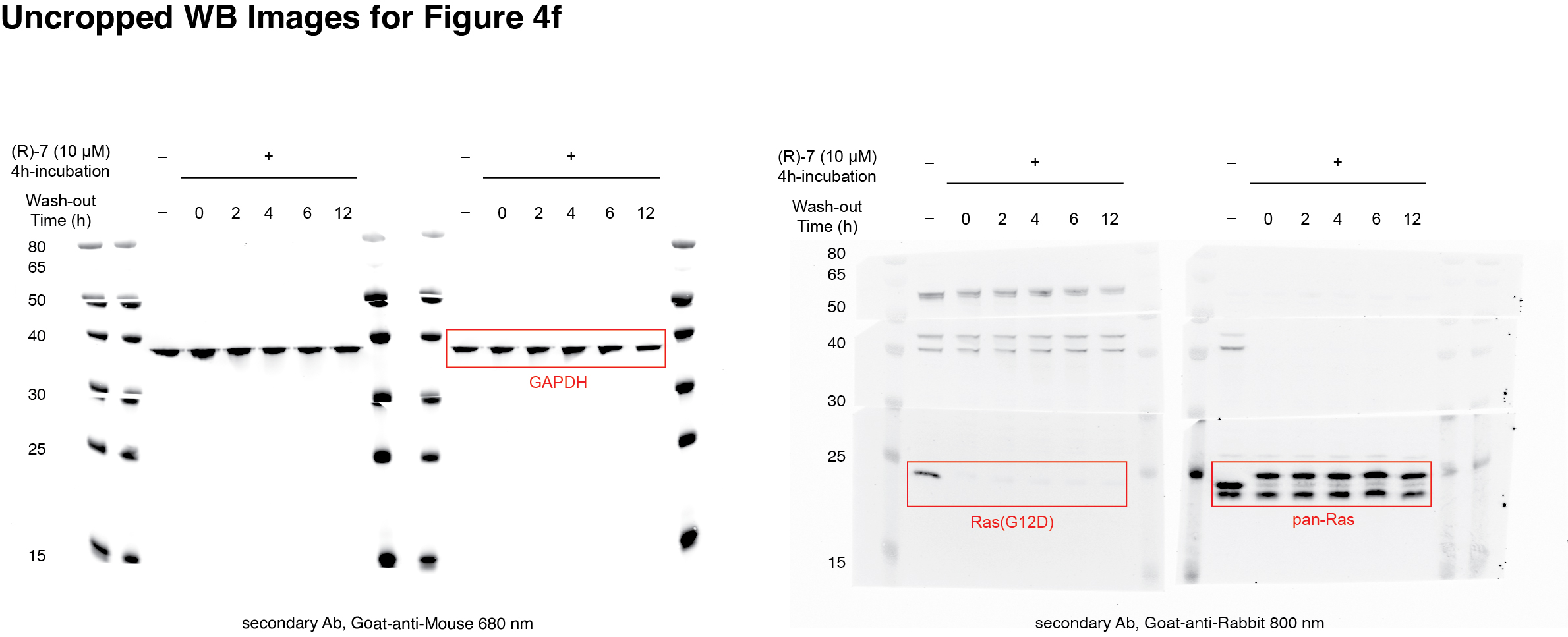

Supplement: Supplementary file 6 — Uncropped western blot images for Fig. 4f. [file 41589_2024_1565_MOESM6_ESM.jpg]

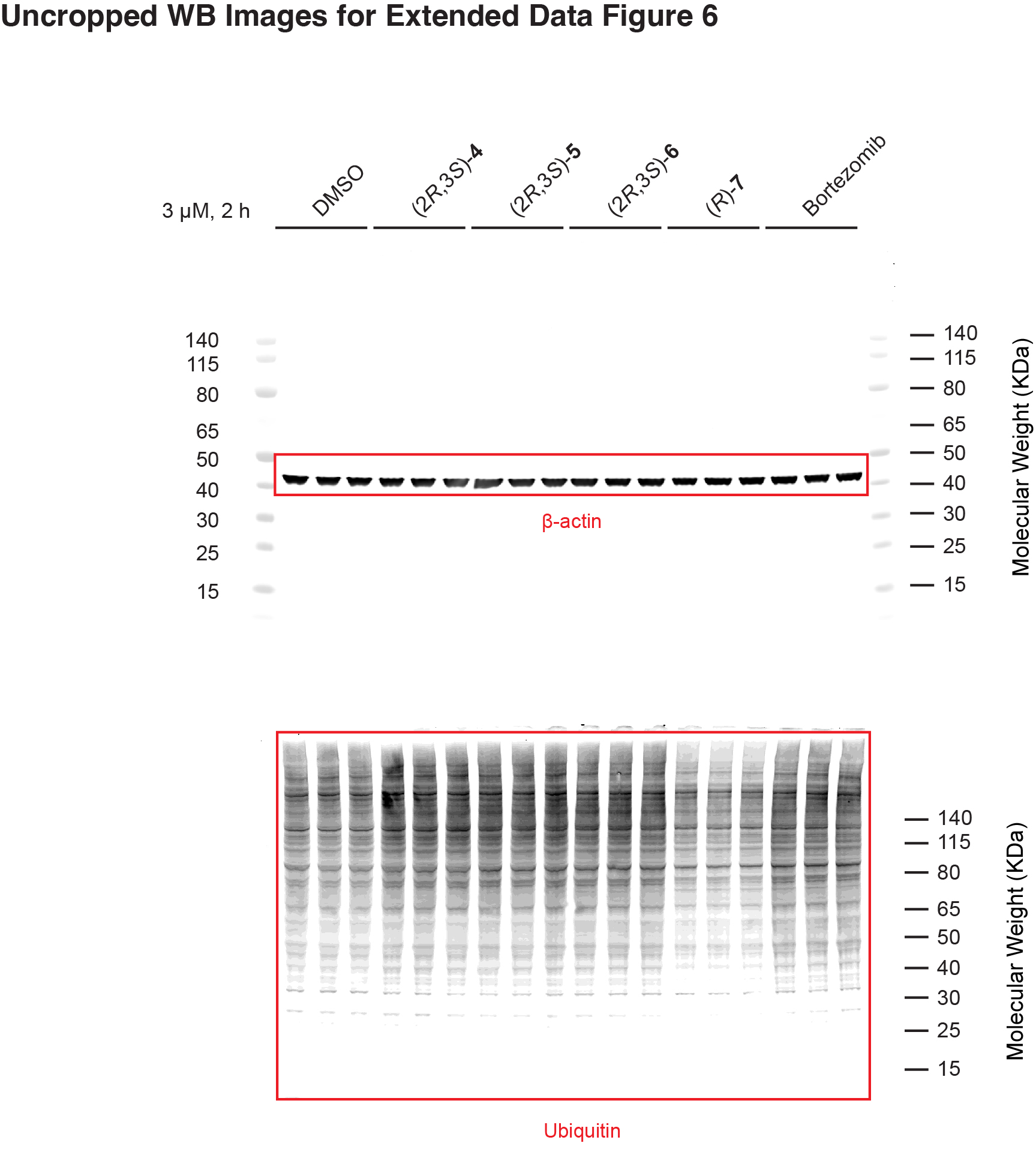

Supplement: Supplementary file 7 — Uncropped western blot images for Extended Data Fig. 6. [file 41589_2024_1565_MOESM7_ESM.jpg]
